# Pharynx associated musculature in the Stilbonematinae (Desmodoroidea, Nematoda) with focus on the spiral muscle as a synapomorphic trait between Dorylaimia and Chromadoria

**DOI:** 10.1007/s13127-025-00687-w

**Published:** 2025-11-14

**Authors:** Philipp Pröts, Jörg A. Ott

**Affiliations:** Department of Functional and Evolutionary Ecology, University of Vienna, Vienna, Austria

**Keywords:** Soft-body morphology, Immunohistochemistry, CLSM, Functional morphology, Phylogenetic relationship, Synapomorphy

## Abstract

Recent molecular phylogenies of Nematoda have suggested a sister-group relationship between Dorylaimia and Chromadoria, but supporting morphological evidence has so far been lacking. Using immunohistochemistry in combination with confocal laser scanning microscopy, we identified pharynx-associated musculature in Stilbonematinae, consisting of buccal dilators, somato-pharyngeal muscles, and a longitudinal spiral muscle encircling nearly the entire pharynx. To trace the evolutionary origin of these structures, we extended our investigation to closely related marine outgroups within Desmodorida but also the basally branching Chromadorida and Enoplia. Somato-pharyngeal muscles, which arise from the body wall musculature and attach to the pharynx surface, function as retractors in Chromadoria and Dorylaimia. In some Enoplia, however, they extend towards the posterior pharynx and act as protractors. These muscles are homologous and thus represent part of the ancestral body plan of Nematoda. Homologous buccal dilators and spiral muscles occur in both Dorylaimia and Chromadoria, whereas in Oncholaimina (Enoplia) buccal dilators—acting as protractors—have evolved independently. Taken together, these morphological findings strongly support a sister-group relationship between Dorylaimia and Chromadoria. A statistical analysis of the Stilbonematinae pharynx and its spiral musculature showed that the number of spiral coils is strongly correlated with pharynx slenderness (length-to-width ratio), in line with Roggen’s pharynx model. In both Dorylaimia and Chromadoria, the spiral musculature surrounding the posterior pharynx likely generates injection pressure, aiding the posterior transport of ingested food into the intestine as well as the anterior movement of secretions from pharyngeal glands.

## Introduction

Our systematic understanding of the phylum Nematoda has changed dramatically with the application of molecular techniques (De Ley & Blaxter, 2004). To date, however, molecular data alone have proven insufficient to robustly resolve the relationships among the three main subclasses of Nematoda: Chromadoria, Enoplia, and Dorylaimia (Schmidt-Rhaesa, 2014). This limitation stems partly from (i) an almost exclusive focus on limnic and terrestrial species, with marine groups—such as Enoplia and the basally branching Chromadoria—largely neglected, and (ii) the low phylogenetic resolution of SSU genes for large-scale analyses (Meldal et al., 2007).

Morphological studies on marine nematodes exist but are restricted to relatively few species and rely on traditional techniques such as light microscopy (LM) and transmission electron microscopy (TEM). Documenting the muscular system in meiofaunal nematodes with these methods is particularly challenging. For instance, the radially symmetric pharynx often requires complete ultrathin sectioning to reconstruct its three-dimensional structure. In small nematodes, semithin sections lack sufficient resolution, while ultrathin sections combined with manual labelling and computational 3D reconstruction are highly time-consuming.

State-of-the-art methods such as immunohistochemical staining of muscles, nuclei, and the nervous system combined with confocal laser scanning microscopy (CLSM) have only recently been applied to Nematoda (Henne et al., 2017; Pröts et al., 2024). Pharynx-associated musculature is rarely reported in species descriptions, partly because these muscles are often delicate, obscured by light refraction, and therefore difficult to observe with LM alone. When reported, such musculature is usually conspicuous and functionally prominent, making it an important taxonomic character (e.g., the “evertor muscles” of *Odontophora falcifera;* Ott, 1972; the “protractor muscles” of *Tylenchus;* Andrássy, 1979; or the “spiral sheath” of *Axonchium;* Coomans & Nair, 1975).

Immunohistochemical labelling offers a more comprehensive view of nematode musculature. While investigating the pharynx of Stilbonematinae using CLSM, we identified several previously unreported pharynx-associated muscles: buccal dilators, anterior and posterior somato-pharyngeal muscles, and a peri-pharyngeal spiral muscle (Pröts et al., 2024). The original aim of this study was to describe and functionally assess the peri-pharyngeal spiral muscle in Stilbonematinae. In addition, where material permitted, we documented and analyzed further pharynx associated muscles.

For clarity, we define these muscle groups as follows:

- 1. *“*Buccal dilator muscles*”* – extending from the anterior pharynx to the anterior body extremity.
- 2. *“*Somato-pharyngeal muscles*”* (after Chitwood & Chitwood, 1977, originally “somato-esophageal muscles”) – divided into anterior and posterior sets. The anterior set originates from the body wall musculature and inserts into the extracellular matrix (ECM) of the anterior pharynx, while the posterior set arises from the somatic musculature and attaches to the posterior bulb.

Unless stated otherwise, descriptions refer to a single body quadrant, which is identical across all four quadrants.

In this study, we document pharynx-associated musculature in Stilbonematinae and investigate its evolutionary origin. To this end, we examined multiple species of Stilbonematinae as well as marine representatives of Desmodorida, Chromadorida, and Enoplia for the presence or absence of these muscles. Our methods combine immunohistochemistry with CLSM and ultrathin sectioning with TEM. Finally, we provide a statistical analysis of the peri-pharyngeal spiral muscle in relation to pharyngeal morphology in Stilbonematinae, supporting the pharynx model proposed by Roggen (1979). The new morphological data presented here for Chromadoria and Enoplia further support a sister-group relationship between Chromadoria and Dorylaimia.

## Materials and methods

### Collection of specimens

Sediment containing *Catanema* sp., *Cyathorobbea hypermnestra* Scharhauser et al., 2024, *Cy. ruetzleri* Scharhauser et al., 2024, *Cy. agricola* Scharhauser et al., 2024, *Robbea judithae* Scharhauser et al., 2024, *Laxus* sp. 1, *L.* sp. 2, and *Paralaxus cocos* Scharhauser et al., 2020 was collected in February 2019 from marine sand near Carrie Bow Cay, Belize. The species *Catanema schiemeri* Ott et al., 2020, *Robbea lotti* Scharhauser et al., 2024, *R. weberae* Scharhauser et al., 2024, *Leptonemella juliae* Hoschitz et al., 1999, *Eubostrichus topiarius* Berger et al., 1996, and *Laxus cosmopolitus* Ott et al., 1995 were extracted from sand collected in the Bay of Sant’Andrea on Elba, Italy, in September 2020. Additional specimens of *Eubostrichus topiarius*, *Leptonemella juliae*, and *Catanema schiemeri*, as well as specimens of Chromadorida (*Chromadorina* sp., *Synonchiella* sp., *Pomponema* sp., *Marylynnia* sp., *Paracyatholaimus* sp.), Desmodorida (*Acanthopharynx* sp., *Paradesmodora* sp., *Desmodora* sp., *Onyx* sp.) and Enoplida (*Mesacanthion* sp., *Enoploides* sp., *Eurystomina* sp., *Thalassironus* sp.) were collected in Veštar, Rovinj, Croatia, in 2022.

### Sample preparation

#### Immunohistochemistry

For immunohistochemistry, specimens were relaxed in MgCl₂ solution isosmotic to seawater. Anterior ends were separated with a razor blade and fixed in 4% paraformaldehyde in 1 × phosphate-buffered saline (PBS, pH 7.3) for 1 h at room temperature (RT). Samples were rinsed three times in PBS (15 min each) and stored at 4 °C in the same buffer until further processing.

Specimens were then treated overnight at RT in 0.1 M phosphate buffer (PB, pH 7.3) containing 1% DMSO and 2% Triton X-100 (PBT). F-actin was stained using Alexa Fluor 488 phalloidin (1:50, Molecular Probes, Eugene, OR, USA) in fresh PBT. After rinsing three times in PBS (15 min each), specimens were mounted on microscope slides in Fluoromount-G (Thermo Fisher Scientific, Waltham, MA, USA). To minimize shrinkage during mounting, Stilbonematinae specimens were embedded in a Fluoromount-G/PBS solution (1:5). Slides were stored at 4 °C in the dark until microscopy and fresh Fluoromount-G was added to the side of the cover glass in case water evaporated.

#### Transmission electron microscopy (TEM)

TEM negatives of *Laxus oneistus, Laxus sp.,* *Catanema schiemeri* and *Cyathorobbea hypermnestra* from the collection of JAO were inverted and analyzed for this study. The original specimens used for TEM were anesthetized in MgCl₂ isosmotic to seawater and fixed following Eisenman and Alfert (1982): 4% glutaraldehyde and 2% osmium tetroxide in 0.1 M sodium cacodylate buffer (pH 7.2). Samples were dehydrated in ethanol and embedded in Spurr’s epoxy resin (Spurr, 1969).

### Image acquisition

Image stacks were obtained using a Leica SP5 confocal laser scanning microscope (Leica Microsystems, Wetzlar, Germany) with LAS AF software. If necessary, background reduction and contrast enhancement were performed in Fiji (RRID:SCR_002285; Schindelin et al., 2012) using the CLAHE module (Zuiderveld, 1994). Volume renderings were generated with Drishti (RRID:SCR_017999; Limaye, 2012), and schematic drawings were created in Inkscape (RRID:SCR_014479).

### Measurements and statistics

To compare two- and three-part pharynges in the context of Roggen’s pharynx model, we quantified the number of muscular coils: in three-part pharynges around the isthmus, and in two-part pharynges around the entire anterior pharynx. For three-part pharynges, isthmus length was measured in full; for two-part pharynges, anterior pharynx length was measured from the first muscle coil to the anterior end of the bulbus.

Measurements were taken from image stacks acquired with LAS AF software and analyzed in Fiji. Muscle coils were counted on one side of the pharynx; in discontinuous spirals, individual muscle braces (spirally arranged longitudinal muscles) were counted as a single coil. Statistical analyses were performed in R (RRID:SCR_001905). Isthmus slenderness was calculated as the ratio of isthmus length to width.

## Results

### Buccal dilator muscles

#### Desmodorida

**Fig. 1:**
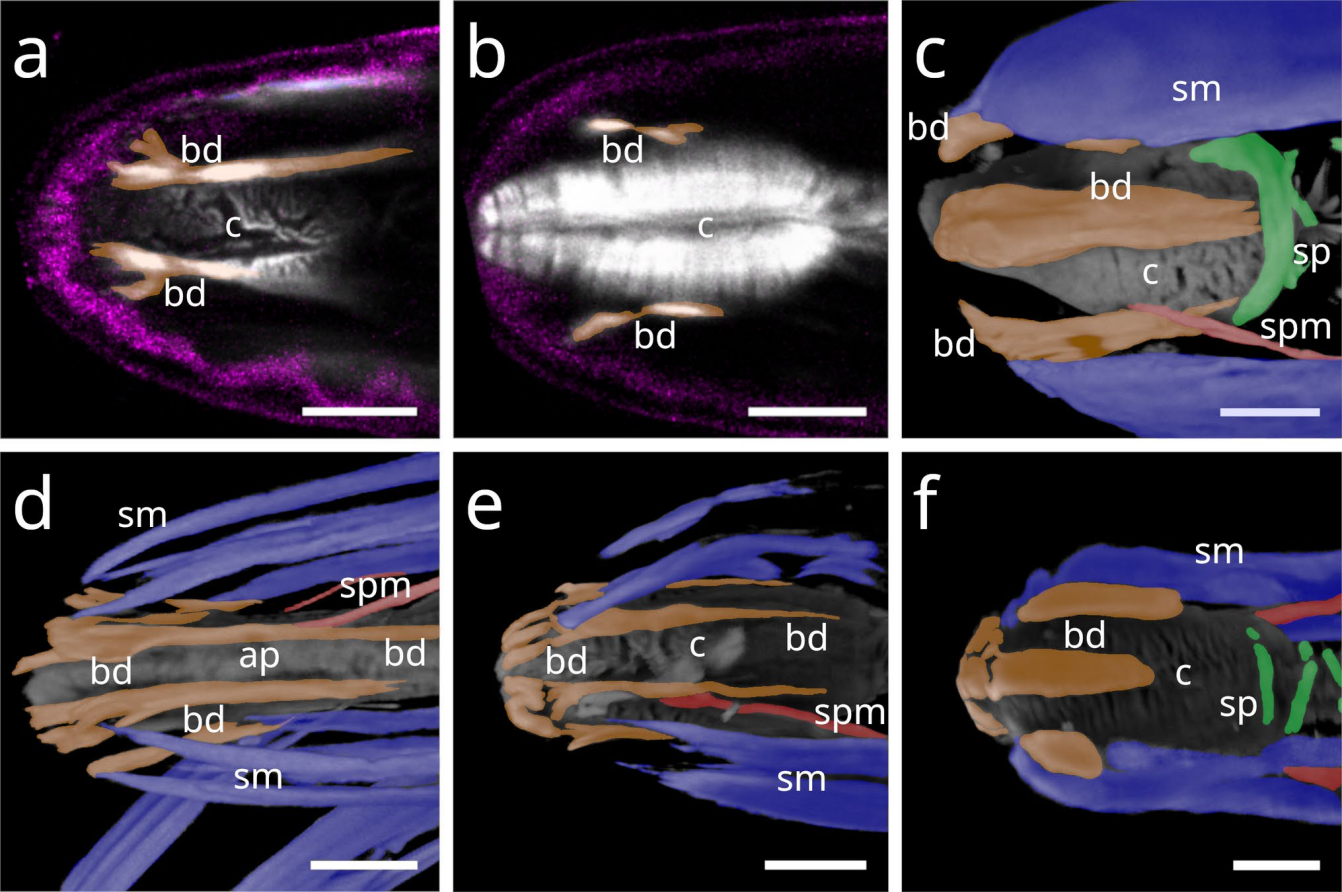
Buccal dilator muscles of Stilbonematinae. CLSM micrographs. Anterior to the left. Buccal dilators in brown, somatic muscles in blue, somato-pharyngeal muscles in red, spiral musculature in green. (**a**) *Catanema schiemeri*. Corpus area. Autofluorescence of cuticle and epidermis in purple. (**b**) *Catanema schiemeri*. Corpus area. (**c**) *Cyathorobbea ruetzleri*. Corpus area. (**d**) *Eubostrichus topiarius*. Anterior-most pharynx end. (**e**) *Robbea judithae*. Corpus area. (**f**) *Robbea judithae*. Corpus area. Abbreviations ap anterior pharynx, bd buccal dilators, c corpus, sm somatic musculature, sp spiral muscle, spm somato-pharyngeal muscle. Scalebars 10 µm

**Fig. 2:**
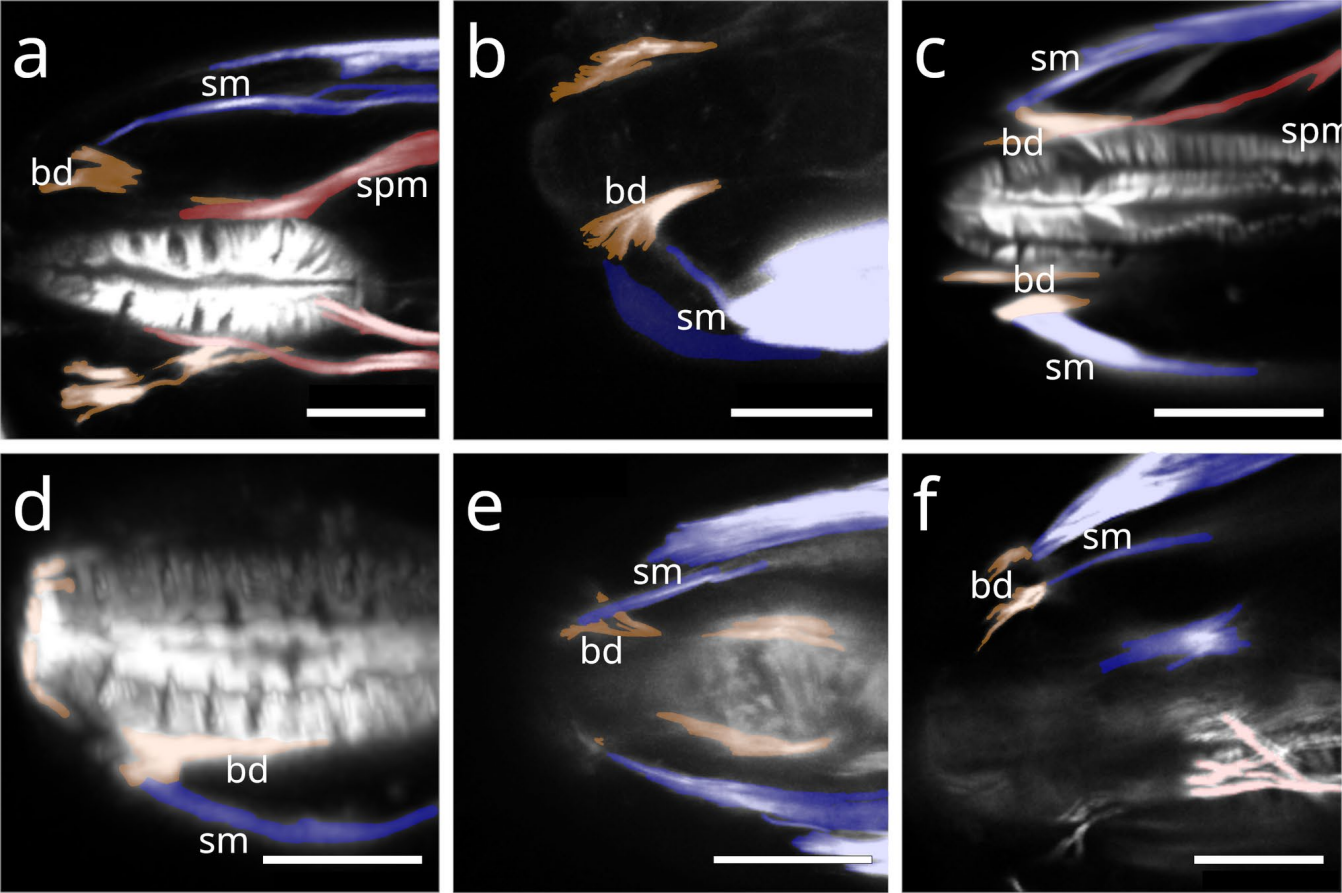
Anterior pharynx, showing the connection of somatic musculature and buccal dilators. Buccal dilators in brown, somatic muscles in blue, somato-pharyngeal muscles in red. Stilbonematine. CLSM micrographs. Anterior to the left. (**a**) *Catanema schiemeri*. **b**) *Cyathorobbea hypermnestra*. (**c**) *Eubostrichus topiarius*. (**d**) *Robbea judithae*. (**e**) *Laxus oneistus*. (**f**) *Laxus cosmopolitus*. Abbreviations bd buccal dilators, sm somatic musculature, spm somato-pharyngeal muscle. Scalebars (a-d, f) 10 µm, (e) 20 µm

In Stilbonematinae (Desmodoridae), the buccal dilator muscles form a set of six that extend from the anterior pharynx to peri-oral sites in dorsal, ventral, subdorsal, and subventral positions. At their anterior ends, they branch and insert at the terminal extremity (Fig. 1 a, b). In some individuals of several species, a direct connection between the somatic musculature and the anterior end of the buccal dilators was observed (Fig. 1 d; Fig. 2 a–f). The phalloidin signal often appeared discontinuous just before the branching point, likely corresponding to the myosin-rich region of the sarcomere. Posteriorly, the muscles attach near the origin sites of the anterior somato-pharyngeal muscles, except for the dorsal and ventral dilators, which lack somato-pharyngeal counterparts (Fig. 1 c,f). This general arrangement was consistent across all Stilbonematinae species examined.

**Fig. 3:**
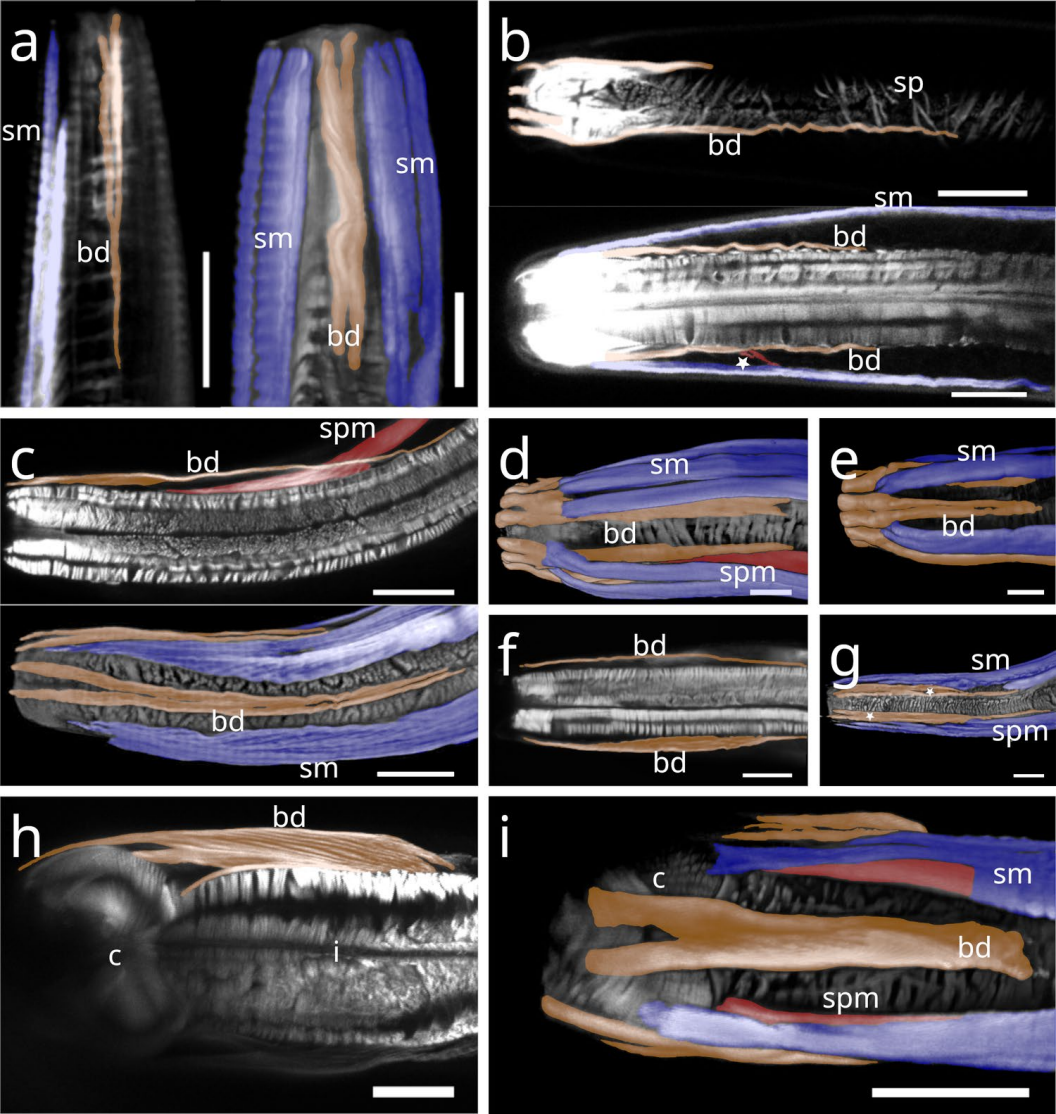
Anterior pharynx showing buccal dilator muscles of Desmodorida and Chromadorida. CLSM micrographs. Buccal dilators in brown, somatic muscles in blue, somato-pharyngeal muscles in red. Anterior to the left, in (a) anterior to the top. (**a**) *Desmodora* sp. (**b**) *Paradesmodora* sp. Asterisk marks a delicate connection of the somatic musculature with the buccal dilator (**c**) *Chromadorina* sp. (**d,e**) *Paracyatholaimus* sp. (**f,g**) *Pomponema* sp. (**h**) *Synonchiella* sp. lateral view. (**i**) *Synonchiella* sp. dorsal view. Abbreviations bd buccal dilator, c corpus, i isthmus, sm somatic musculature, sp spiral muscle, spm somato-pharyngeal muscle. Scalebars (a-f, h) 20 µm (g,i) 40 µm

In *Desmodora* sp. (Desmodoridae, Desmodorinae), six buccal dilators extend from approximately mid-pharynx to the anterior extremity. Their posterior ends form a single muscle bundle, which bifurcates near the anterior extremity (Fig. 3 a).

In *Paradesmodora* sp. (Desmodoridae, Spiriniinae), the six buccal dilators extend from about one-third of total pharynx length to the perioral region (Fig. 3 b; 8 b). On the subdorsal and subventral sides, delicate myofilaments from the somatic musculature connect to these muscles. Available material of *Acanthopharynx* sp., *Spirinia* sp., and *Onyx* sp. did not allow unambiguous identification of buccal dilators.

#### Chromadorida

In *Chromadorina* sp. (Chromadoridae, Chromadorinae), the buccal dilators originate slightly posterior to the point where somato-pharyngeal muscles arise from the somatic musculature. They extend from the pharynx to the perioral region (Fig. 3 c). On the dorsal and ventral sides, no connection with the somatic musculature was observed. The subdorsal and subventral dilators, however, rise from the pharynx surface and fuse with the somatic musculature before continuing anteriorly. The anterior parts split into two branches attaching at the terminal extremity, whereas the remainder of the somatic muscles connect to the pharynx slightly more posteriorly (Fig. 3 c).

In *Paracyatholaimus* sp. (Cyatholaimidae, Paracanthonchinae), buccal dilators extend from the pharynx near the attachment sites of the anterior somato-pharyngeal muscles to the anterior extremity (Fig. 3 d,e). The anterior third of the buccal dilators merge with somatic musculature en route; the dorsal and ventral muscles are joined by both the respective subdorsal and subventral somatic muscles (Fig. 23).

In *Pomponema* sp. (Cyatholaimidae, Pomponematinae), the buccal dilators extend from about mid-pharynx to the perioral extremity. Each splits into two equally developed branches anteriorly (Fig. 3 f,g). The subdorsal and subventral dilators are joined by myofilaments of the somatic musculature.

In *Synonchiella* sp. (Selachinematidae), the attachment of buccal dilators and anterior somato-pharyngeal muscles is complex and asymmetric. Both muscle groups arise from the somatic musculature and attach to the anterior corpus, with the buccal dilators continuing forward to the perioral extremity (Fig. 3h, i). The buccal dilators start at about the anterior third of the pharynx and extend to the anterior pharynx and head extremity. Just anterior to their posterior ends, the lateral-most part of the somatic musculature fuses with the subdorsal and subventral dilators, except for the left subventral one. Likewise, dorsal and ventral dilators showed no apparent connection.

Attachment patterns vary by position:

- *Dorsal side***:** A single muscle splits into a delicate flat proximal branch attached to the pharynx surface and a distal branch. The distal branch travels along the proximal surface, then elevates and divides into four, with two remaining proximal (attaching at mid-corpus and its base) and two extending to the anterior extremity.
- *Subdorsal sides***:** Each muscle splits into two, travels along the pharynx surface, elevates, and extends to the anterior extremity. Anterior somatic muscle fibres split into two bundles, one attaching to the corpus at mid-length and the other (i.e. the somato-pharyngeal muscle) at the corpus base.
- *Ventral side***:** The dilators splits posteriorly into two branches that extend to the anterior extremity. No attachments at mid-corpus or corpus base were observed.
- *Left subventral side***:** Represented by a flat longitudinal muscle lacking somatic connections, extending from the pharynx surface to the corpus base, resembling the flat proximal dorsal branch of the right subventral side (see below). No attachments to anterior extremity or mid-corpus were present.
- *Right subventral side***:** The muscle splits into a flat proximal branch attaching to the corpus base and a distal branch that divides into three anteriorly—two reaching the anterior extremity and one attaching at mid-corpus. Somatic musculature inserts adjacent to the mid-corpus attachment.

#### Enoplida

**Fig. 4:**
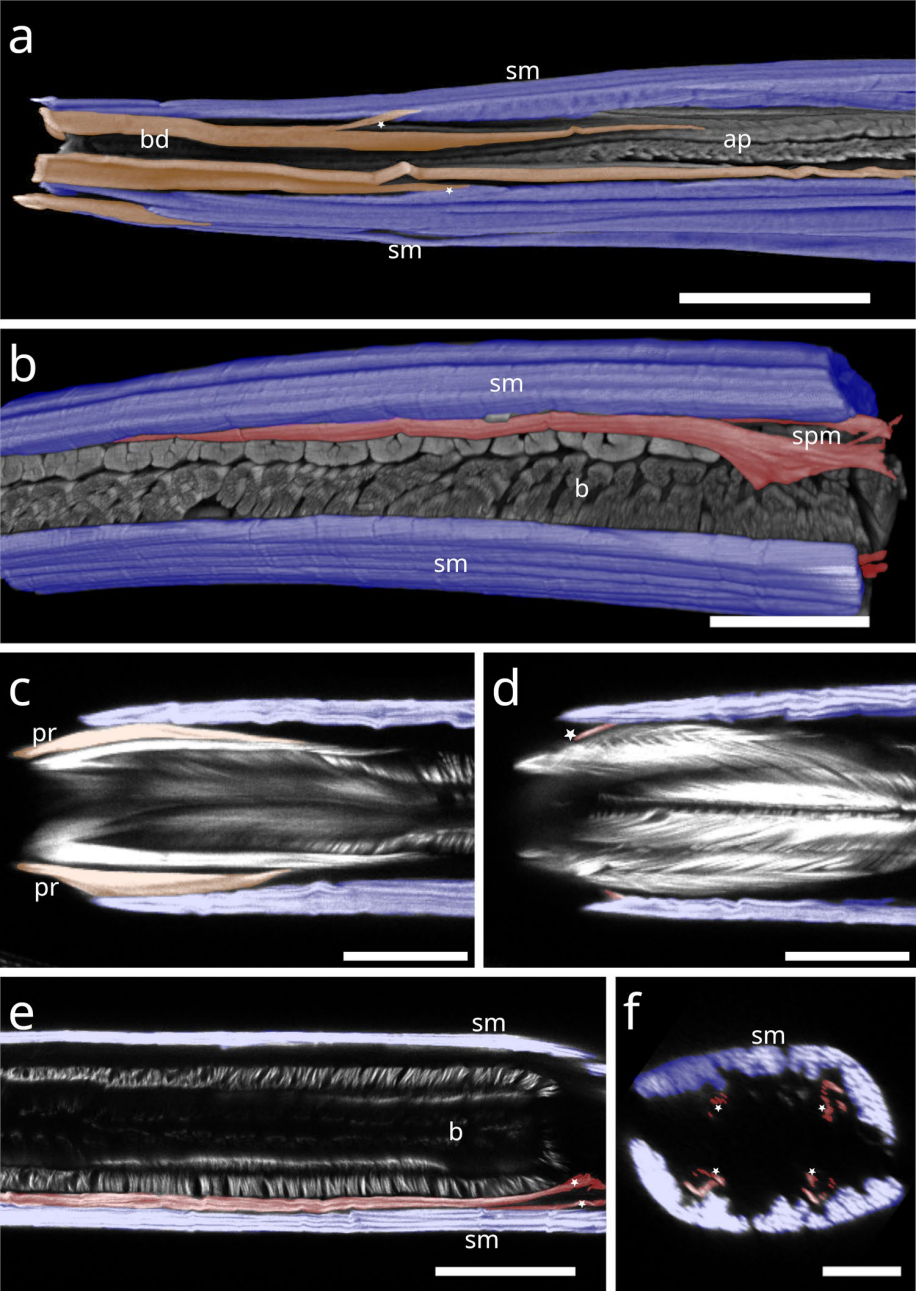
Buccal dilators and somato-pharyngeal muscles of Enoplida. CLSM micrographs. Buccal dilators in brown, somatic muscles in blue, somato-pharyngeal muscles in red. Anterior to the left. (**a**) *Eurystomina* sp. Anterior pharynx. (**b**) *Eurystomina* sp. Posterior pharynx. (**c**) *Thalassironus* sp. Protractor muscles. (**d**) *Thalassironus* sp. Anterior somato-pharyngeal muscles. (**e**) *Thalassironus* sp. Posterior somato-pharyngeal muscles. (**f**) *Thalassironus* sp. Posterior somato-pharyngeal muscles in optical cross section. Abbreviations ap anterior pharynx, b base of the pharynx, bd buccal dilators, pr protractor muscle, sm somatic musculature, spm somato-pharyngeal muscle, tm tooth muscle. Asterisks in (a) connection of somatic musculature with buccal dilators, (d) anterior somato-pharyngeal muscle, (e, f) posterior somato-pharyngeal muscles. Scalebars (a, b, e) 40 µm, (c, d, f) 20 µm

In *Eurystomina* sp. (Oncholaimina, Enchelidiidae), *seven buccal dilators* were observed: one dorsal, two subdorsal, two subventral, and *two distinct ventral muscles*. They extend from the anterior pharynx to the perioral area, becoming more prominent anteriorly. At mid-length, delicate myofilaments of the somatic musculature fuse with the dilators (Fig. 4 a). Somatic filaments from both left and right subdorsal bundles join the dorsal dilator. On the ventral side each muscle is joined by the respective subventral somatic musculature (Fig. 23).

In *Thalassironus* sp. (Enoplina, Ironidae, Thalassironinae), a single protractor muscle is present per pharyngeal sector—one dorsal and two subventral (Fig. 4 c). Each is flanked by two tooth muscles within the same sector. The protractors extend beyond the remaining anterior pharyngeal musculature to the lip bases.

**Fig. 5:**
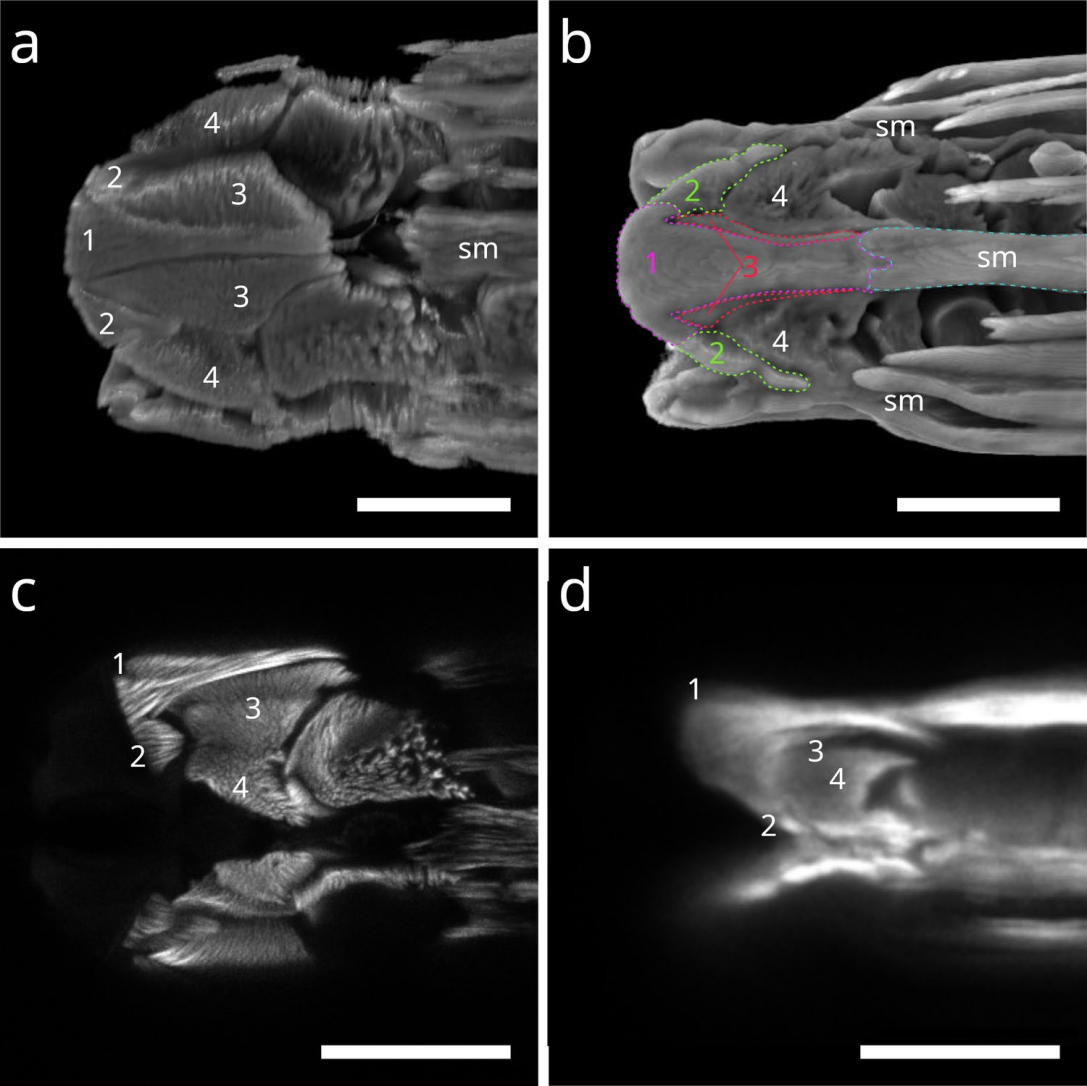
Homologous anterior pharynx muscles of Enoplida. CLSM micrographs. Anterior to the left. (**a**, **c**) *Enoploides* sp. (**b**,**d**) *Mesacanthion* sp. Abbreviations sm somatic musculature. Scalebars (a) 40 µm, (b-d) 20 µm

Similarly, in *Enoploides* sp. and *Mesacanthion* sp. (Enoplina, Thoracostomopsidae, Enoplolaiminae), the anterior pharynx exhibits the typical Enoplid radial muscle complex, i.e. the pharynx is fused with the buccal cavity. On the dorsal and subventral sides, the pharyngeal sectors are bent outwards anteriorly. Elongated pharyngeal muscles (Fig. 5 a-d; muscle “1”) are flanked by several additional radial muscles (muscles “2,” “3,” and “4”) and extend from the pharyngeal ECM to the base of the onchial plates of the buccal cavity.

In *Mesacanthion* sp., somatic musculature attaches dorsally, ventrally, subdorsally, and subventrally to the anterior pharynx. On the dorsal and subventral sides, it connects to muscle “1” at about one-third of its posterior length. In contrast, in *Enoploides*, the somatic musculature terminates posterior to these muscles and does not attach to the complex (Fig. 5 a–d).

### Somato-pharyngeal muscles

The somato-pharyngeal musculature is defined here as an anterior and a posterior set of typically four longitudinal muscles (one per body quadrant). The myofilaments are continuous with portions of the body wall musculature, bending towards and ultimately attaching to the pharyngeal surface. The anterior set is associated with the anterior pharynx, whereas the posterior set connects directly to the posterior bulbus. Unless otherwise noted, the following descriptions refer to a single body quadrant.

#### Desmodorida

**Fig. 6:**
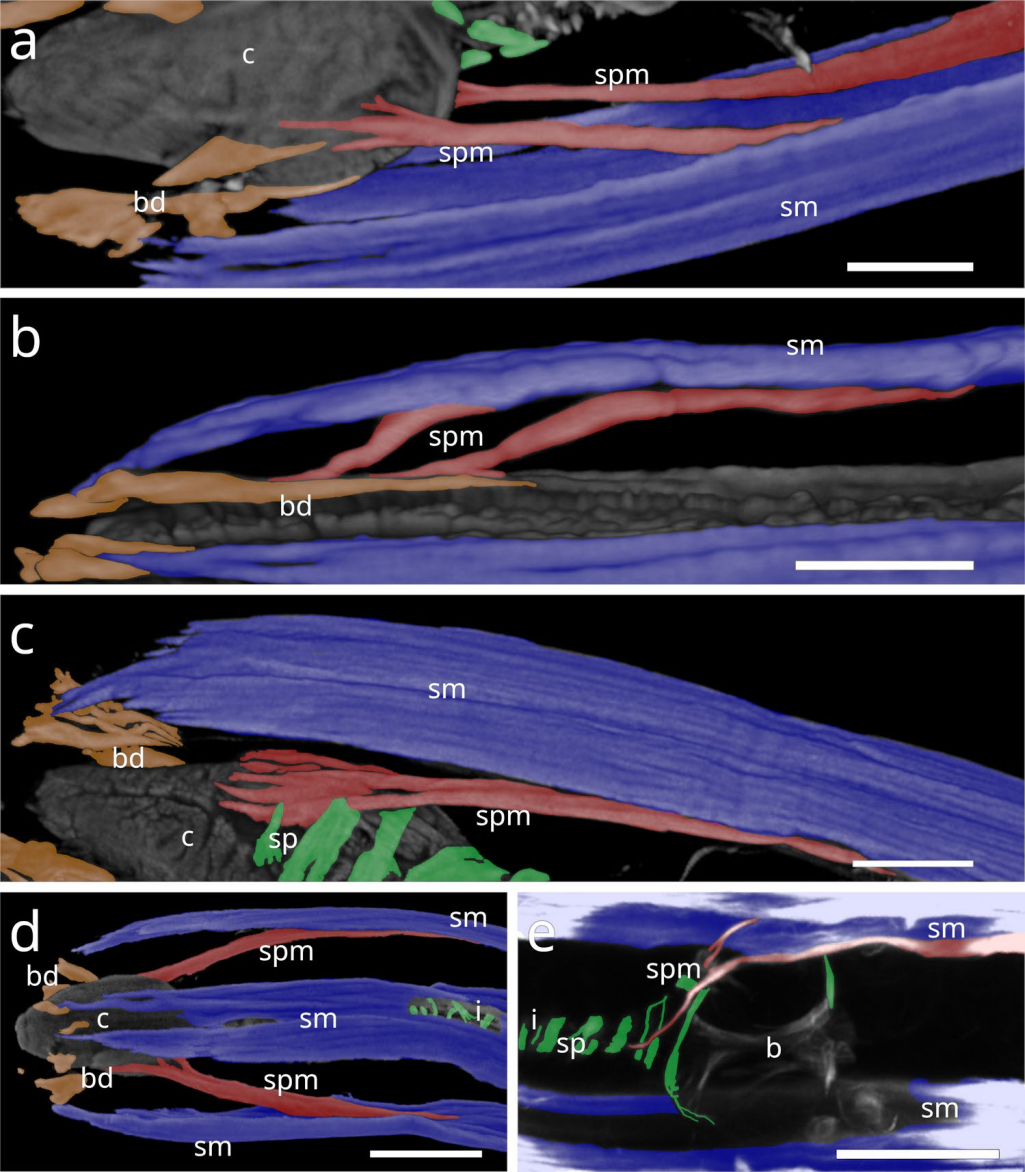
Somato-pharyngeal muscles of Stilbonematinae. CLSM micrographs. Buccal dilators in brown, somatic muscles in blue, somato-pharyngeal muscles in red, spiral musculature in green. Anterior to the left. (**a**) *Cyathorobbea ruetzleri*. (**b**) *Eubostrichus topiarius*. (**c**) *Laxus oneistus*. (**d**) *Catanema schiemeri*. (**e**) *Catanema schiemeri*. Bulbus area. Abbreviations b bulbus, bd buccal dilators, c corpus, i isthmus, sm somatic musculature, sp spiral muscle, spm somato-pharyngeal muscle. Scalebars (a,c,d,e) 20 µm (b) 10 µm

**Fig. 7:**
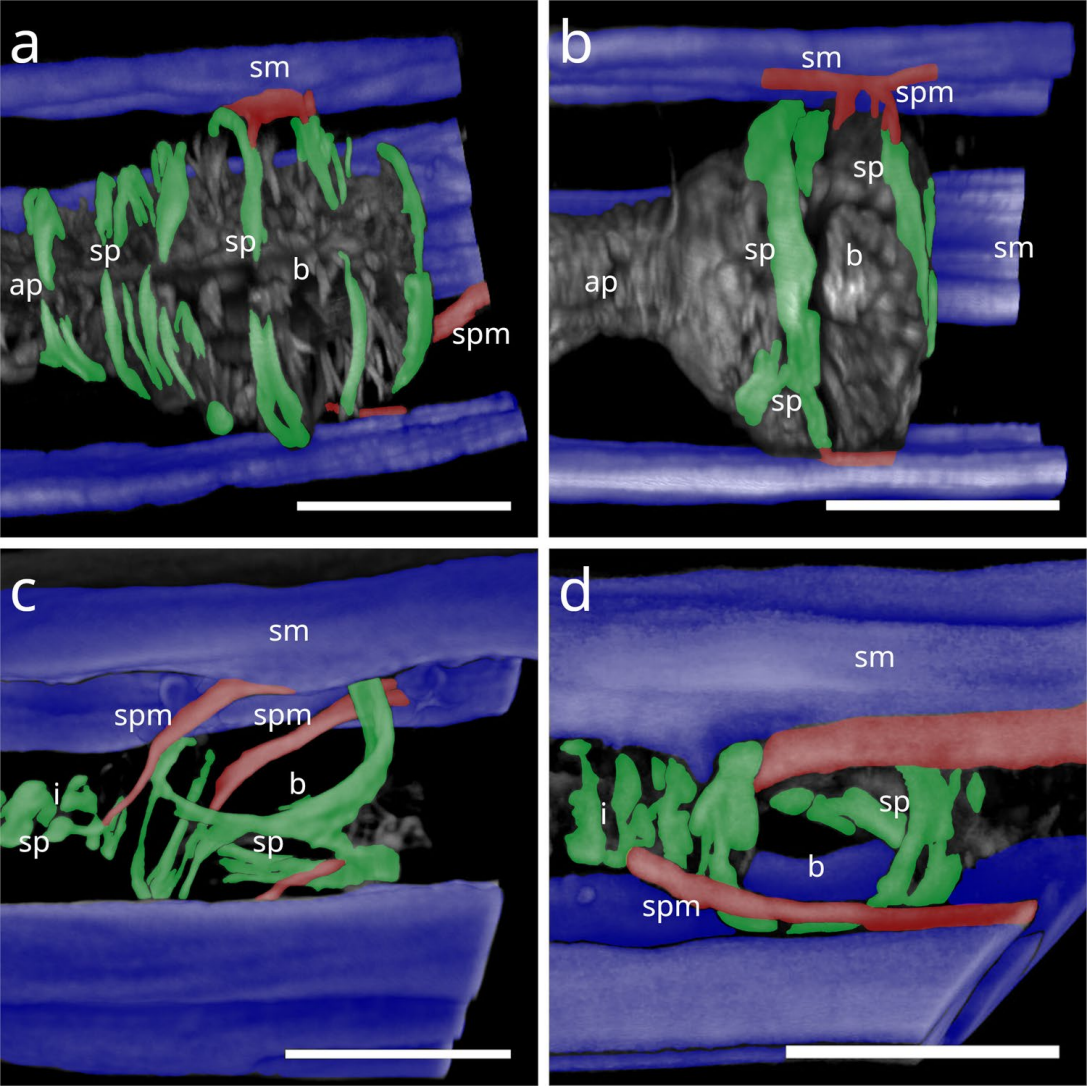
CLSM micrographs of spiral musculature of Stilbonematinae. Bulbus area. Somatic muscles in blue, somato-pharyngeal muscles in red, spiral musculature in green. Anterior to the left. (**a**) *Leptonemella juliae*. (**b**) *Eubostrichus topiarius*. (**c**) *Catanema schiemeri*. (**d**) *Catanema* sp. Abbreviations ap anterior pharynx, b bulbus, i isthmus, sm somatic musculature, sp spiral muscle, spm somato-pharyngeal muscle. Scalebars 20 µm

In Stilbonematinae, at approximately half the pharynx length, the anterior somato-pharyngeal muscles bend towards and attach to the anterior pharyngeal surface (Fig. 1 c–e; Fig. 6 a–d). This condition was observed in all examined Stilbonematinae species, except *Eubostrichus topiarius*, which possesses an additional muscle that bends towards the anterior pharynx (Fig. 6 b). The posterior somato-pharyngeal muscles originate from the body wall musculature at the level of the bulbus, where they attach (Fig. 6 e; Fig. 7 a–d). These posterior muscles are short, except in *Catanema*, *Cyathorobbea hypermnestra, Laxus oneistus, Laxus* sp. 1 and *Paralaxus cocus*, where they are elongated and insert at the anterior end or the mid part of the bulbus (Fig. 6 e; 7 c,d; 11 b; 14 a,c).

**Fig. 8:**
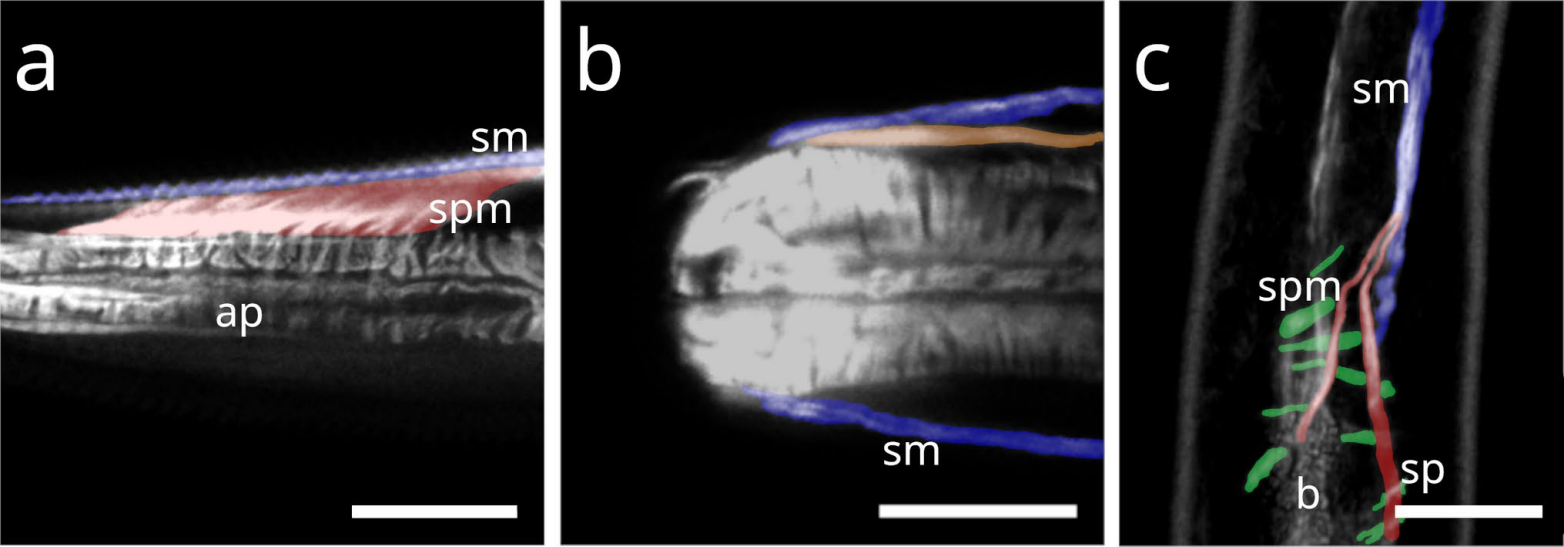
Somato-pharyngeal muscles of Desmodorida. CLSM micrographs. Buccal dilators in brown, somatic muscles in blue, somato-pharyngeal muscles in red, spiral musculature in green. Anterior to the left in (**a,b**) Anterior to the top in (**c**). (a) *Desmodora* sp. mid-part of the anterior pharynx. (b) *Paradesmodora* sp. Anterior pharynx. (c) *Paradesmodora* sp. Posterior pharynx area. Abbreviations ap anterior pharynx, b base of the pharynx, sm somatic musculature, sp spiral muscle, spm somato-pharyngeal muscle. Scalebars 20 µm

In *Desmodora* sp., anterior somato-pharyngeal muscles are present in the form of a series of muscles in the first half of the pharynx, in subventral position. They consist of diagonal myofilaments that extend from the somatic musculature to the pharynx in anterior direction (Fig. 8 a). These connect to the pharyngeal surface near the subventral buccal dilators. The outer margin of the posterior bulb is in slight contact with the somatic musculature, but no distinct posterior somato-pharyngeal muscles were observed.

In *Paradesmodora* sp., the somatic musculature does not give rise to separate anterior somato-pharyngeal muscles but instead connects directly to the anterior pharynx (Fig. 6 b). The posterior somato-pharyngeal muscles arise from the somatic musculature at about the last fourth of the pharynx length and extend towards the bulbus, where they appear to transition into the spiral musculature (Fig. 8 c).

In *Acanthopharynx* and *Onyx* image data were insufficient to determine the presence or absence of these muscles.

#### Chromadorida

**Fig. 9:**
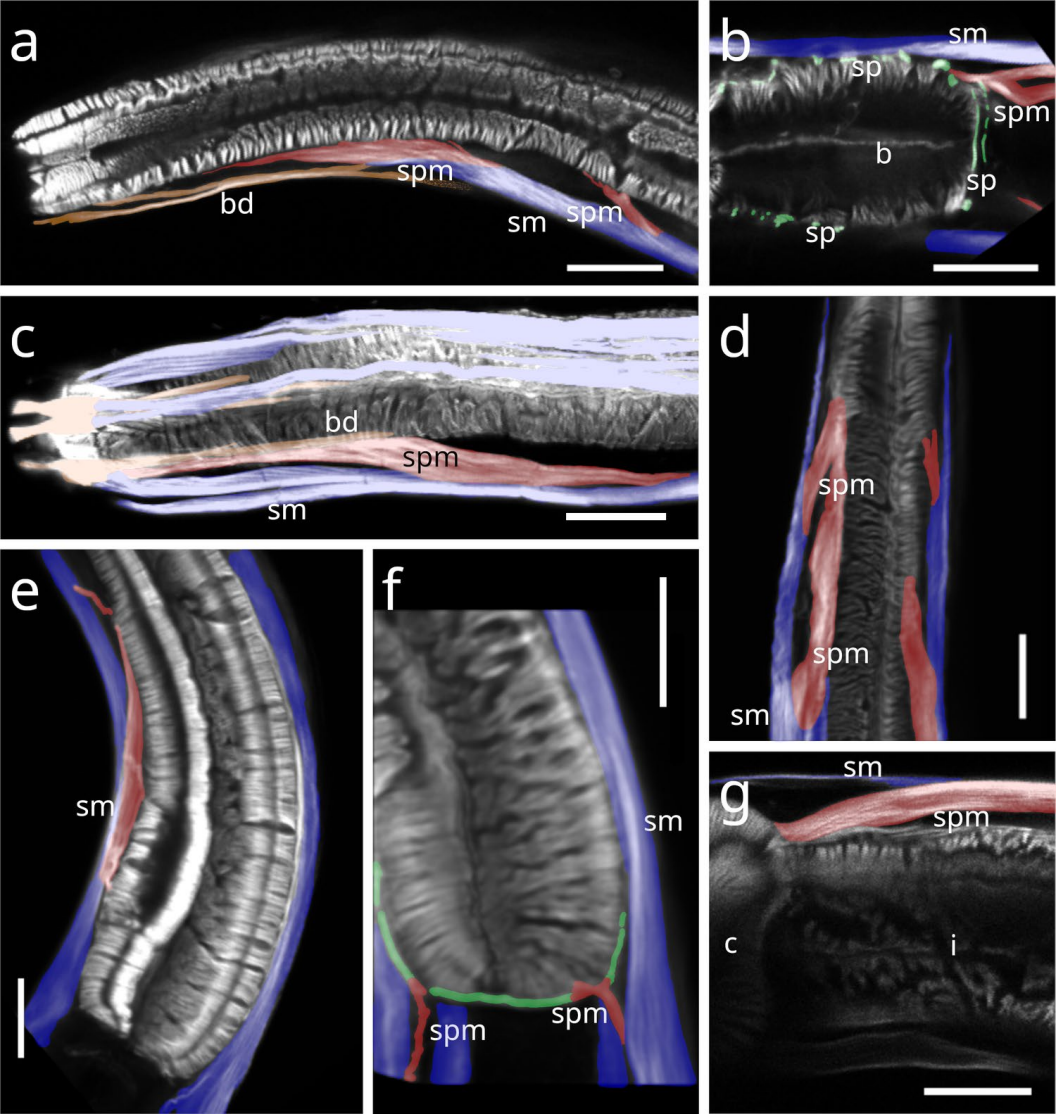
Somato-pharyngeal muscles of Chromadorida. CLSM micrographs. Buccal dilators in brown, somatic muscles in blue, somato-pharyngeal muscles in red, spiral musculature in green. Anterior to the left. (**a**) *Chromadorina* sp. Anterior pharynx. (**b**) *Chromadorina* sp. Posterior pharynx. (**c**) *Paracyatholaimus* sp. Anterior pharynx. (**d**) *Pomponema* sp. Anterior to the top. Anterior pharynx (**e**) *Pomponema* sp. Posterior pharynx. (**f**) *Pomponema* sp. Posterior-most pharynx end. (**g**) *Synonchiella* sp. Corpus-isthmus area. Abbreviations ap anterior pharynx, b base of the pharynx, c corpus, i isthmus, sm somatic musculature, sp spiral muscle, spm somato-pharyngeal muscle. Scalebars 20 µm

In *Chromadorina* sp., two anterior somato-pharyngeal muscles originate from the somatic musculature. At about half the pharynx length, the somatic musculature splits into two branches: one inserts into the pharyngeal surface, while the other continues anteriorly and gives rise to a second somato-pharyngeal muscle at about one third of the pharynx length (Fig. 9 a). The posterior somato-pharyngeal muscles arise from the somatic musculature posterior to the bulbus and connect to its posterior end (Fig. 9 b).

In *Paracyatholaimus* sp., at approximately two-thirds of the pharynx length, the somatic musculature gives rise to somato-pharyngeal muscles that turn proximally and extend along the anterior pharynx, adjacent to the buccal dilators (Fig. 3 d; 9 c). No posterior set was observed; instead, the posterior pharynx is in direct contact with the somatic musculature.

In *Pomponema* sp., several sets of somato-pharyngeal muscles are present. At about one third of the pharynx length, part of the somatic musculature gives rise to two additional muscles. The proximal muscle bends towards the pharynx and extends anteriorly along its surface, while the distal branch follows the somatic musculature for a short distance before bending anteriorly and fusing with the proximal muscle on the pharynx surface (Fig. 9 d). Further posteriorly, near the level of the posterior pharynx, delicate somato-pharyngeal muscles connect to the pharynx (Fig. 9 e). Short posterior somato-pharyngeal muscles also extend from the subdorsal and subventral somatic musculature towards the posterior pharynx end (Fig. 9 f).

In *Synonchiella* sp., the anterior somato-pharyngeal muscles originate slightly posterior to the distinctly set-off corpus, bending towards the pharynx and attaching at the base of the corpus on the subdorsal sides and at mid-corpus on the subventral sides (Fig. 9 g). No posterior set was observed. The somatic musculature also joins the buccal dilators (see the chapter about the buccal dilators for details).

#### Enoplida

In *Eurystomina* sp., only distinct posterior somato-pharyngeal muscles are present. They arise from the somatic musculature at about the last third of the pharynx length, bifurcate and extend posteriorly to the end of the pharynx (Fig. 4 b).

In *Thalassironus* sp., a short anterior somato-pharyngeal muscle originates from the somatic musculature and connects to the anterior pharynx (Fig. 4 d). A posterior set is extending from the body wall musculature posteriorly, past the base of the posterior pharynx, and likely connecting to the anterior intestine in vivo (Fig. 4 e,f).

In *Enoploides* sp. and *Mesacanthion* sp., no somato-pharyngeal muscles were detected.

### Spiral muscle

#### Stilbonematinae

**Fig. 10:**
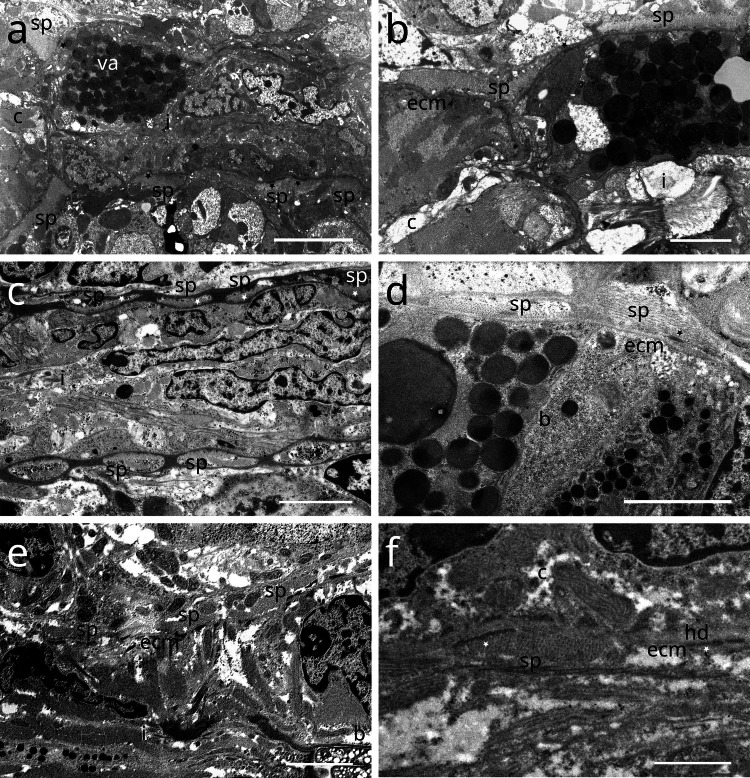
Spiral muscle of Stilbonematinae. TEM micrographs. (**a**) *Cyathorobbea hypermnestra*. Anterior isthmus in the region of the subventral ampulla. (**b**) Detail of (a). (**c**) *Catanema schiemeri*. Isthmus. Cross section of neighbouring coils of the spiral muscle around the isthmus. (**d**) *Catanema schiemeri*. Distal bulbus area surrounded by spiral muscle. (**e**) *Laxus* sp. Isthmus-bulbus transition surrounded by spiral muscle. (**f**) *Laxus oneistus*. Corpus with adjacent spiral muscle. Asterisks indicate hemidesmosomes. Abbreviations b bulbus, c corpus, ecm extracellular matrix, sp spiral muscle, va subventral ampulla. Scalebars (a + e) 5 µm, (b-d) 2.5 µm, (f) 1 µm

**Fig. 11:**
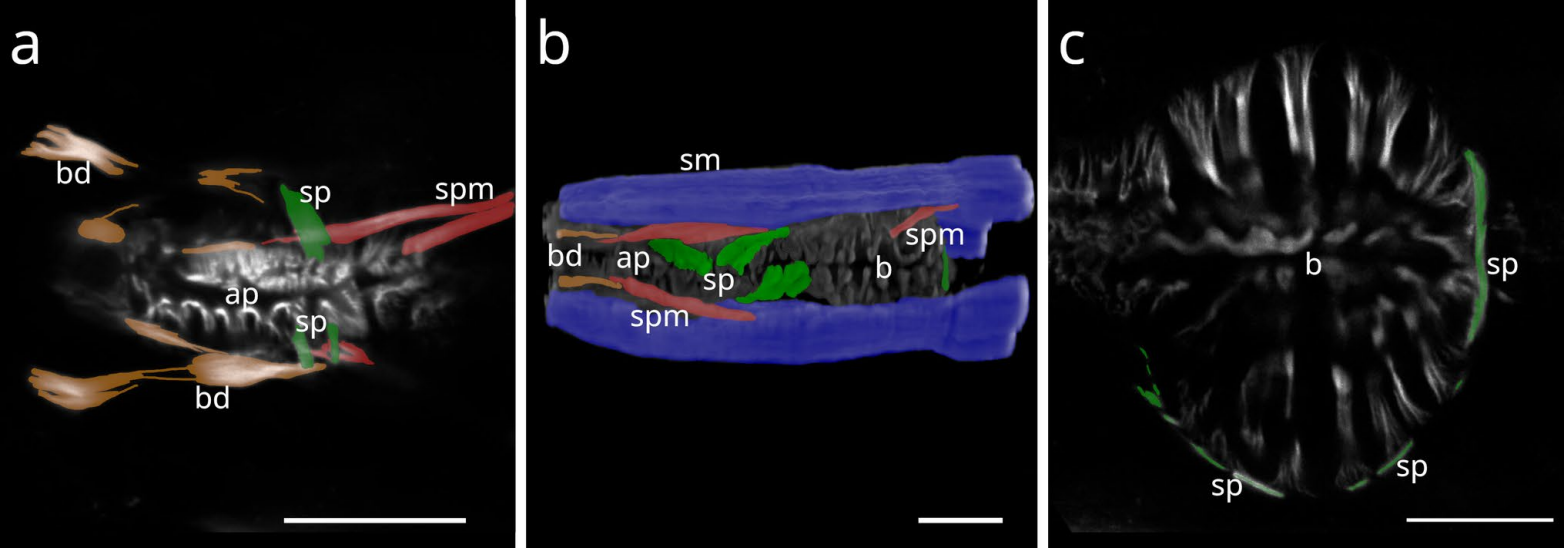
Spiral musculature of *Paralaxus cocos*. CLSM micrographs. Buccal dilators in brown, somatic muscles in blue, somato-pharyngeal muscles in red, spiral musculature in green. Anterior to the left. (**a**) Anterior pharynx area. (**b**) Whole pharynx. (**c**) Bulbus area. Strong phalloidin signal at the distal margin of the bulbus indicate cross sections of the adjacent spiral muscle. Abbreviation ap anterior pharynx, b bulbus, bd buccal dilators, sm somatic musculature, sp spiral muscle, spm somato-pharyngeal muscle. Scalebars (a,c) 15 µm (b) 20 µm

**Fig. 12:**
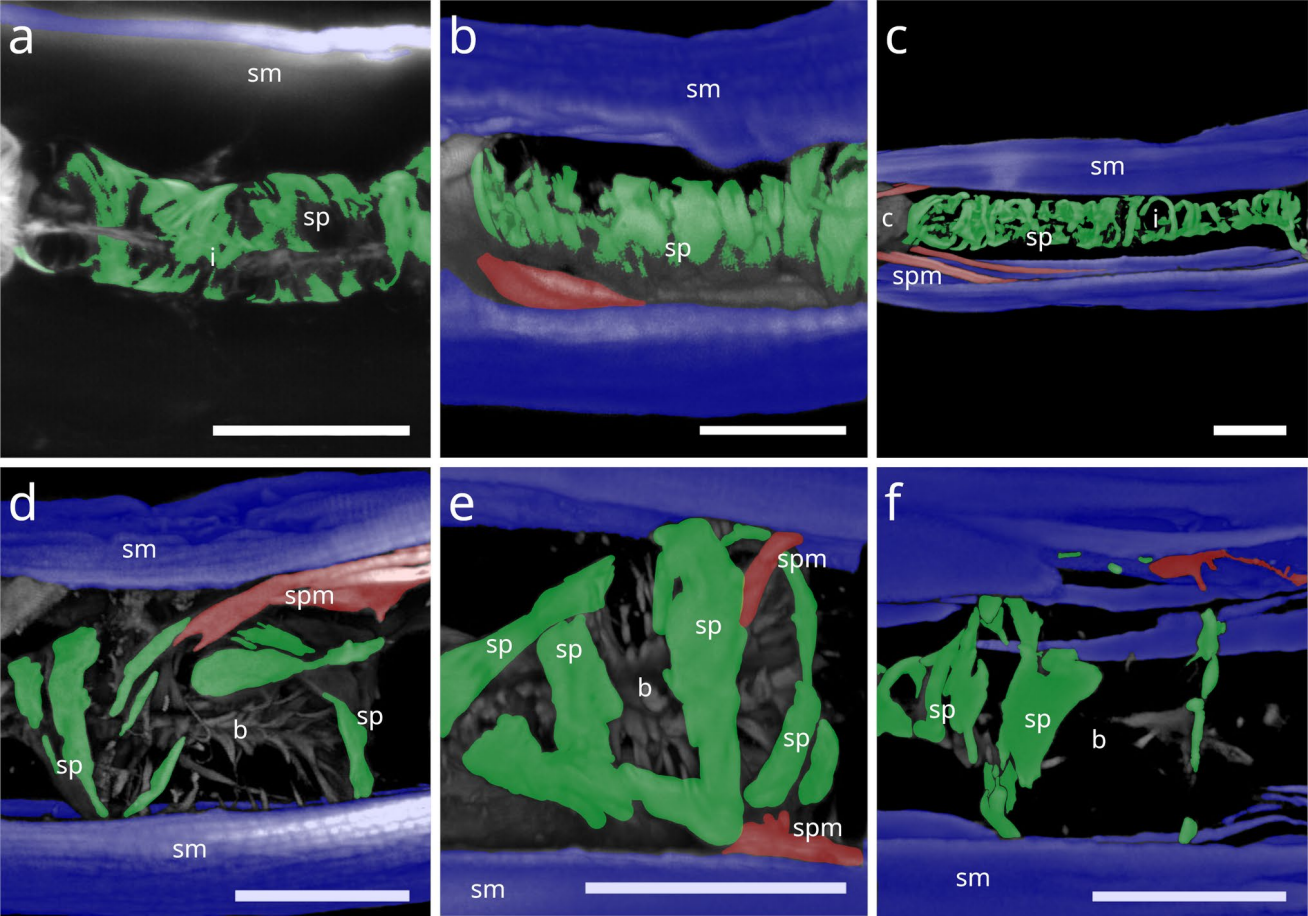
Spiral musculature of the genus *Cyathorobbea* (Stilbonematinae). CLSM micrographs. Somatic muscles in blue, somato-pharyngeal muscles in red, spiral musculature in green. Anterior to the left. (**a**) *Cyathorobbea hypermnestra*. Isthmus. (**b**) *Cyathorobbea ruetzleri*. Isthmus. (**c**) *Cyathorobbea agricola*. Isthmus. (**d**) *Cyathorobbea hypermnestra*. Bulbus. (**e**) *Cyathorobbea ruetzleri*. Bulbus. (**f**) *Cyathorobbea agricola*. Bulbus. Abbreviations b bulbus, c corpus, i isthmus, sm somatic musculature, sp spiral muscle, spm somato-pharyngeal muscle. Scalebars 20 µm

**Fig. 13:**
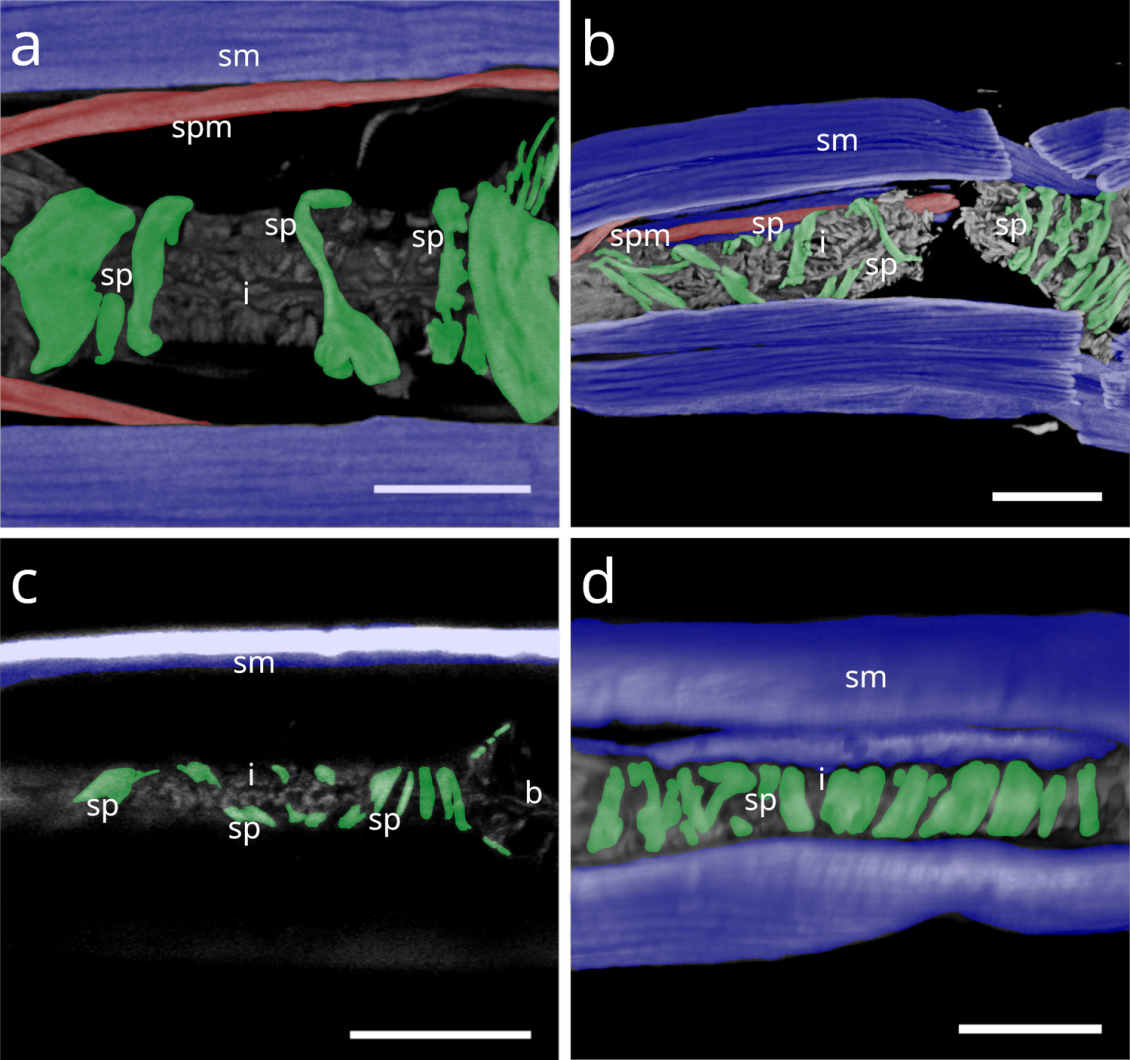
Spiral musculature of the genus *Laxus* (Stilbonematinae). CLSM micrographs. Isthmus area. Somatic muscles in blue, somato-pharyngeal muscles in red, spiral musculature in green. Anterior to the left. (**a**) *Laxus oneistus*. (**b**) *Laxus cosmopolitus*. (**c**) *Laxus* sp.1 (**d**) *Laxus* sp.2. Abbreviations b bulbus, i isthmus, sm somatic musculature, sp spiral muscle, spm somato-pharyngeal muscle. Scalebars 20 µm

**Fig. 14:**
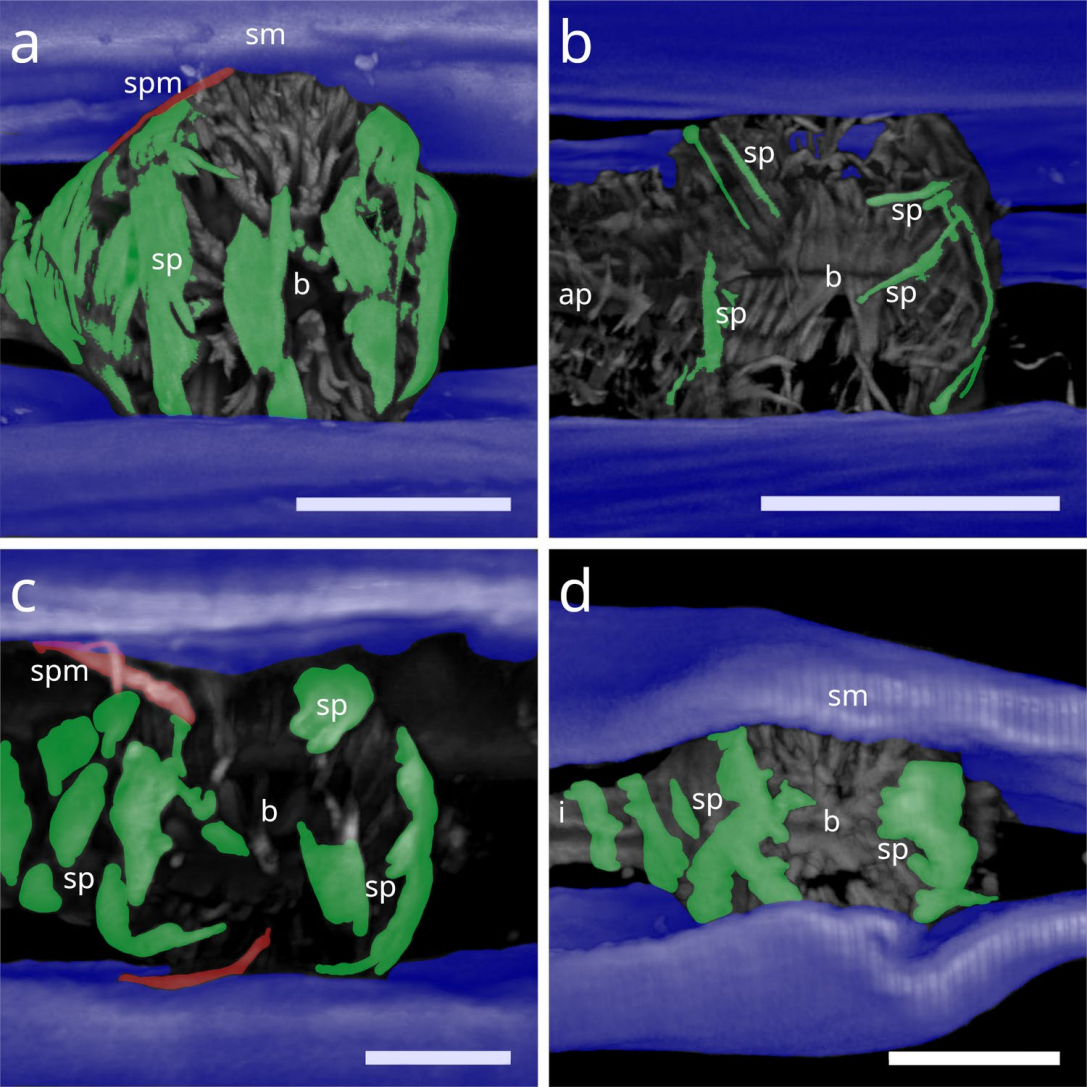
Spiral musculature of the genus *Laxus*. CLSM micrographs. Bulbus area. Anterior to the left. (**a**) *Laxus oneistus*. (**b**) *Laxus cosmopolitus*. (**c**) *Laxus* sp.1. (**d**) *Laxus* sp.2. Abbreviations ap anterior pharynx, b bulbus, i isthmus, sm somatic musculature, sp spiral muscle, spm somato-pharyngeal muscle. Scalebars (a,b,d) 20 µm, (c) 10 µm

**Fig. 15:**
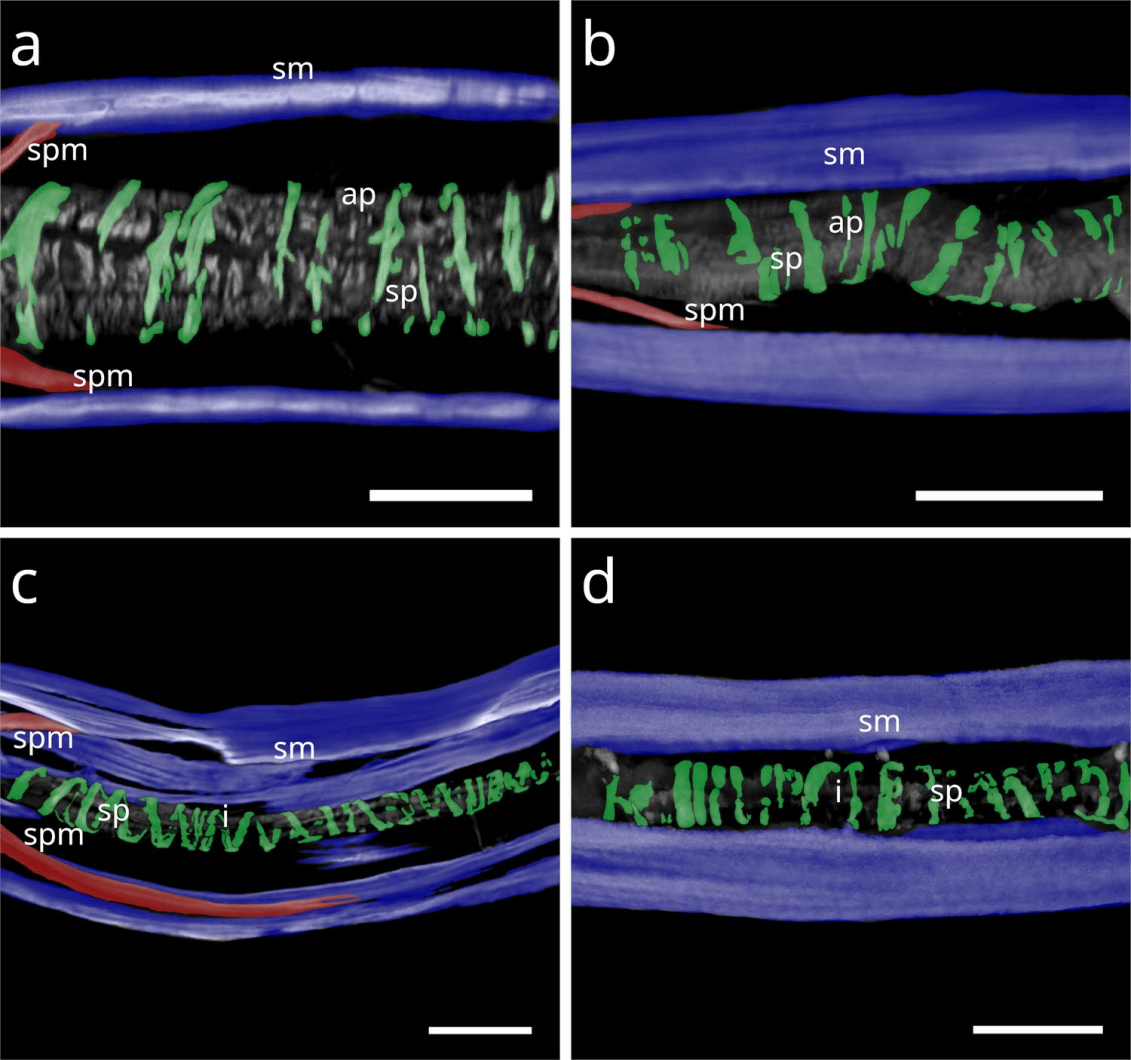
Spiral musculature around the anterior pharynx/isthmus in Stilbonematinae. CLSM micrographs. Somatic muscles in blue, somato-pharyngeal muscles in red, spiral musculature in green. Anterior to the left. (**a**) *Leptonemella juliae*. (**b**) *Eubostrichus topiarius*. (**c**) *Catanema schiemeri*. (**d**) *Catanema* sp. Abbreviations ap anterior pharynx, i isthmus, sm somatic musculature, sp spiral muscle, spm somato-pharyngeal muscle. Scalebars 20 µm

**Fig. 16:**
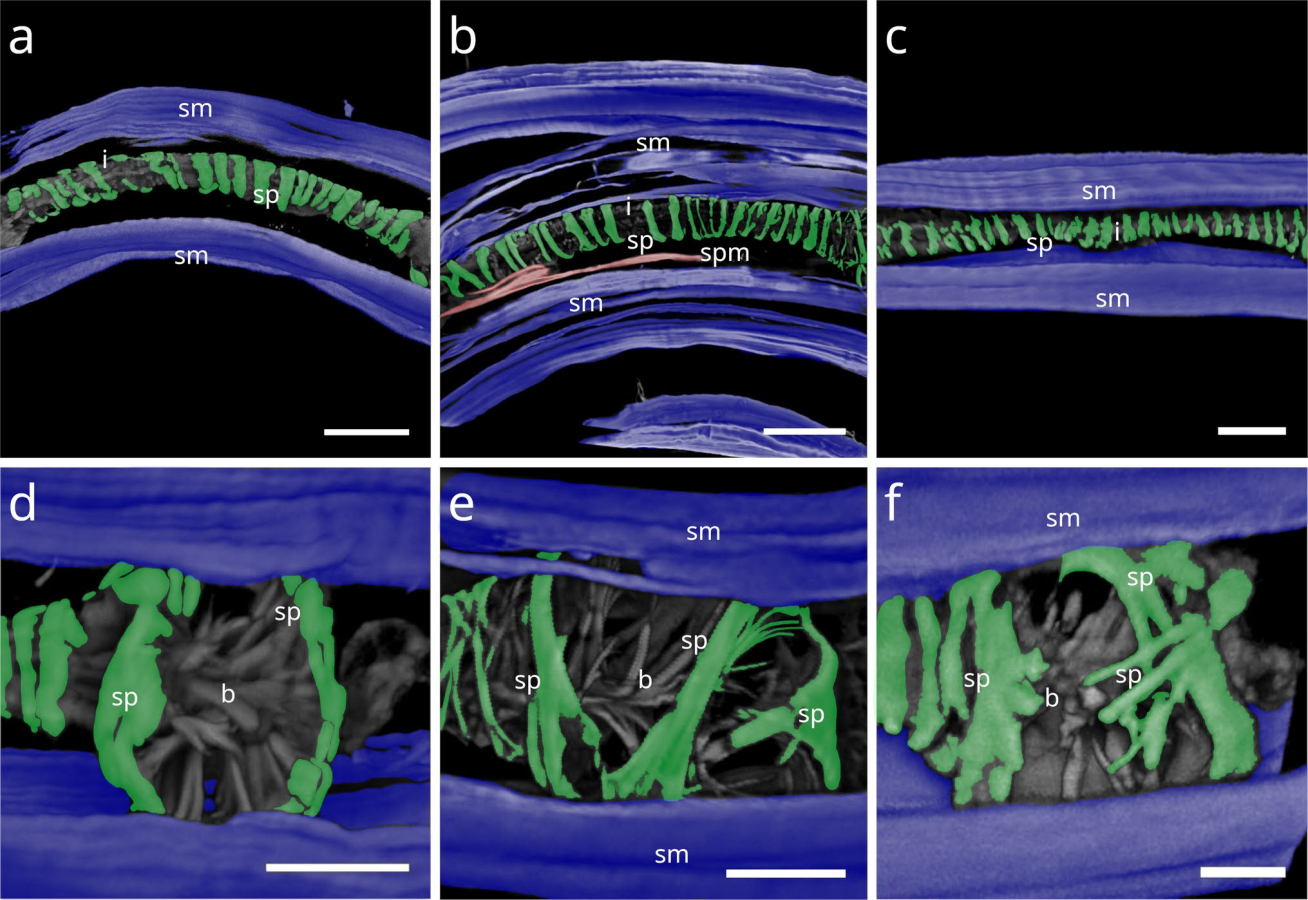
Spiral musculature of the genus *Robbea* (Stilbonematinae). CLSM micrographs. Somatic muscles in blue, somato-pharyngeal muscles in red, spiral musculature in green. Anterior to the left. (**a**) *Robbea judithae*. Isthmus. (**b**) *Robbea lotti*. Isthmus. (**c**) *Robbea weberae*. Isthmus. (**d**) *Robbea judithae*. Bulbus. (**e**) *Robbea lotti*. Bulbus. (**f**) *Robbea weberae*. Bulbus. Abbreviations b bulbus, i isthmus, sm somatic musculature, sp spiral muscle, spm somato-pharyngeal muscle. Scalebars (a-c) 20 µm (d-f) 10 µm

**Fig. 17:**
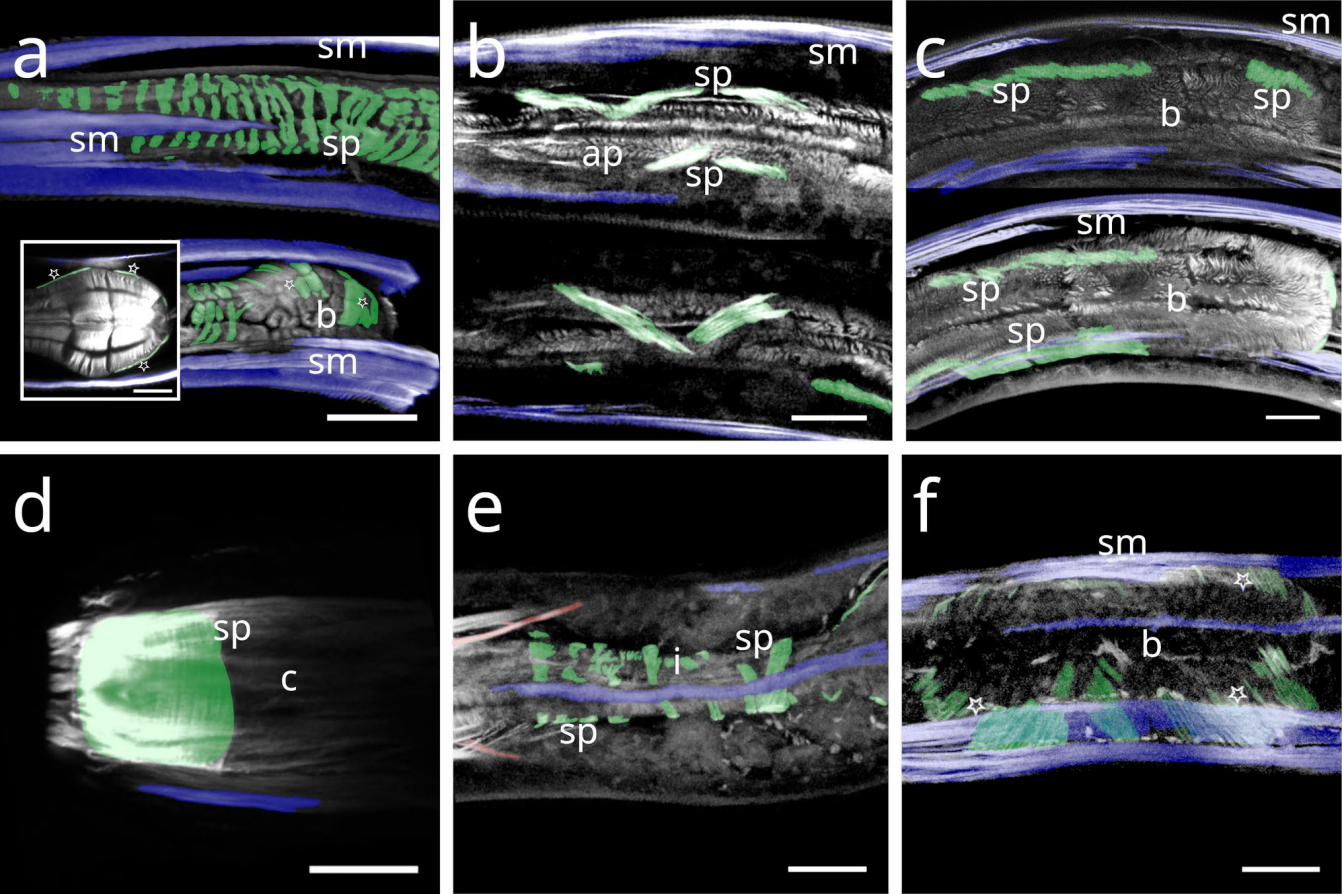
Spiral musculature of Desmodorida. CLSM micrographs. Somatic muscles in blue, somato-pharyngeal muscles in red, spiral musculature in green. Anterior to the left. (**a**) *Desmodora* sp. Anterior pharynx at the top, posterior pharynx at the bottom, cross section of the bulbus in the box. (**b**) *Acanthopharynx* sp. Anterior pharynx. (**c**) *Acanthopharynx* sp. Posterior pharynx. (**d**) *Onyx* sp. Corpus area. (**e**) *Onyx* sp. Isthmus area. (**f**) *Onyx* sp. Bulbus area. Abbreviations ap anterior pharynx, b bulbus, c corpus, i isthmus, sm somatic musculature, sp spiral muscle, asterisks further indicate spiral muscles. Scalebars (a-f) 20 µm, box in (a) 10 µm

In Stilbonematinae, the pharyngeal spiral muscle coils around the basal side of the ECM of the radial musculature (Fig. 10 a–f). It consists of longitudinal myofilaments that extend from the anterior pharynx and encircle both the isthmus and posterior bulb (Fig. 10–17). In genera such as *Catanema*, *Robbea*, and *Laxus*, the corpus is prominently swollen and partly surrounded by the spiral muscle. The spiral muscles are thin, ranging from 0.5–2.5 µm in thickness and are anchored to the pharynx ECM via hemidesmosomes (Fig. 10 a–f). For simplicity, the number of muscular coils is grouped into three categories: (i) 6–10 = “few”, (ii) 11–15 = “intermediate”, (iii) 16 + = “many”. Measurements are summarized in Table 1.

**Table 1:** Measurement data used for statistical analysis of the spiral muscle of Stilbonematinae species.”Isthmus index [length/width]” is the slenderness of the isthmus

Individual	Isthmus length [µm]	Isthmus index [length/width]	Coils around isthmus	Coils around isthmus rank	Isthmus index [length/width] rank	Isthmus length rank
*Cyathorobbea hypermnestra*	46.43	2.77	7	3.5	1	7
*Paralaxus cocos*	41.97	3.13	7	3.5	2	4
*Laxus oneistus*	36.73	3.16	4	1	3	1
*Paralaxus cocos*	42.65	3.83	6	2	4	5
*Laxus cosmopolitus*	49.85	4.14	8	4.5	5	10
*Cyathorobbea ruetzleri*	59.54	4.19	8	4.5	6	15
*Laxus sp.1 (“berm”)*	64.21	4.37	8	4.5	7	20
*Laxus cosmopolitus*	37.12	5.05	8	4.5	8	3
*Eubostrichus topiarius*	61.05	5.13	10	5.5	9	16
*Leptonemella juliae*	47.55	5.25	10	5.5	10	9
*Laxus cosmopolitus*	37.02	5.27	8	4.5	11	2
*Laxus cosmopolitus*	64.17	5.57	8	4.5	12	19
*Cyathorobbea ruetzleri*	52.01	6.02	8	4.5	13	11
*Leptonemella juliae*	66.41	6.33	10	5.5	14	22
*Eubostrichus topiarius*	54.35	7.15	14	8.5	15	14
*Eubostrichus topiarius*	54.03	7.44	12	6.5	16.5	13
*Leptonemella juliae*	70.19	7.44	8	4.5	16.5	27
*Stilbonema majum*	101.94	7.45	13	7	18	32
*Catanema sp. (“schuppe”)*	64.86	7.94	23	12	19	21
*Laxus sp. 2 (“tief”)*	45.99	8.17	12	6.5	20	6
*Cyathorobbea agricola*	53.03	8.47	17	9	21	12
*Catanema schiemeri*	68.31	8.7	22	11.5	22	25
*Catanema schiemeri*	73.52	9.04	21	10	23	29
*Robbea judithae*	62.81	9.13	22	11.5	24	17
*Eubostrichus topiarius*	66.65	9.32	14	8.5	25	24
*Eubostrichus topiarius*	64.08	9.34	14	8.5	26	18
*Robbea lotti*	87.19	9.5	22	11.5	27	30
*Robbea lotti*	66.52	9.84	25	14.5	28	23
*Catanema schiemeri*	94.09	10.15	27	15	29	31
*Eubostrichus topiarius*	46.84	11.55	14	8.5	30	8
*Robbea lotti*	69.27	11.68	25	14.5	31	26
*Robbea weberae*	73.15	13.18	34	16	32	28
*Catanema schiemeri*	102.1	17.94	24	13	33	33

In *Paralaxus cocos*, the pharynx is stout and bipartite, consisting of a short cylindrical anterior portion and a prominent spherical bulbus. The anterior pharynx is surrounded by three delicate myofilaments that serve as attachment sites for the somato-pharyngeal muscles (Fig. 11 a). Posterior to these, the spiral musculature consists only of brace-like myofilaments, a few of which partially surround the mid-pharynx and extend to the posterior isthmus (Fig. 11 b). The bulbus is evenly encircled by several brace-like myofilaments (Fig. 11 c).

The tripartite pharynx of *Cyathorobbea* species varies in overall length, proportions of subparts, and the number of spiral coils. In all species examined, the spiral musculature begins at the posterior end of the corpus, with interruptions visible along its length. *Cyathorobbea hypermnestra* and *Cy. ruetzleri* possess few brace-like coils around the stout isthmus, which are closely arranged and occasionally overlapping (Fig. 12 a,b). By contrast, *Cy. agricola* has many coils encircling its long, slender isthmus (Fig. 10 c). In all species, the spiral muscle continues to wrap around the posterior bulb (Fig. 12 d–f).

In *Laxus*, the number of brace-like coils around the isthmus ranges from few to intermediate. *L. oneistus*, *L. cosmopolitus*, and *L.* sp. 1 possess a stout isthmus with few coils (Fig. 13 a–c), whereas *L.* sp. 2 has an intermediate number surrounding the slender isthmus (Fig. 13 d). In *L. oneistus* and *L.* sp. 2, which possess a swollen corpus, the anterior spiral musculature begins at about mid-corpus. Around the bulbus, *L. oneistus* has broad brace-like coils (Fig. 14 a), *L. cosmopolitus* has loosely arranged delicate coils (Fig. 14 b) around the anterior and posterior part of the bulbus, and *L.* sp. 1 has loosely arranged but broader coils (Fig. 14 c). In *L.* sp. 2, the myofilaments are broad and appear continuous, wrapping both the anterior and posterior ends of the bulbus but leaving a distinct gap at mid-bulbus (Fig. 14 d).

In *Leptonemella juliae*, few muscle coils surround the stout anterior pharynx (Fig. 15 a). The spiral muscle begins slightly posterior to the anterior somato-pharyngeal attachment sites and continues along the pharynx (Fig. 7 a; 15 a).

In *Eubostrichus topiarius*, the number of coils ranges from few to intermediate around the anterior pharynx (Fig. 15 b). The spiral musculature is present around the posterior bulb in all specimens observed (Fig. 7 b).

In *Catanema*, the spiral muscle begins in the posterior quarter of the corpus and continues along the pharynx. In both *C. schiemeri* and *Catanema* sp., the isthmus is evenly encircled (Fig. 15 c,d). Around the bulbus, the myofilament orientation shifts from transverse to nearly parallel with the longitudinal axis (Fig. 7 c,d).

In *Robbea*, the spiral muscle begins at the posterior corpus end, is densely arranged, and consists of many coils covering the entire pharynx (Fig. 16 a–c). In *R. judithae*, spiral musculature of the bulbus is restricted to its anterior and posterior ends (Fig. 16 d), whereas in *R. lotti* and *R. weberae*, the bulbus is loosely encircled (Fig. 16 e,f).

#### Desmodorida

In *Desmodora* sp., the pharynx (except for its extreme anteriormost end) is wrapped in a sheath of many continuous coils (Fig. 17 a). The coils around the posterior portion are not continuous.

In *Acanthopharynx* sp., few brace-like muscles encircle the anterior pharynx (Fig. 17 b), and a few series of short myofilaments partially wrap the bulbus (Fig. 17 c).

In *Onyx* sp., the corpus is encased in a hull of muscles (Fig. 17 d), the isthmus is surrounded by few continuous coils (Fig. 17 e), and the posterior bulb is wrapped in a loosely arranged basket-like sheath of delicate coils (Fig. 17 f).

#### Chromadorida

**Fig. 18:**
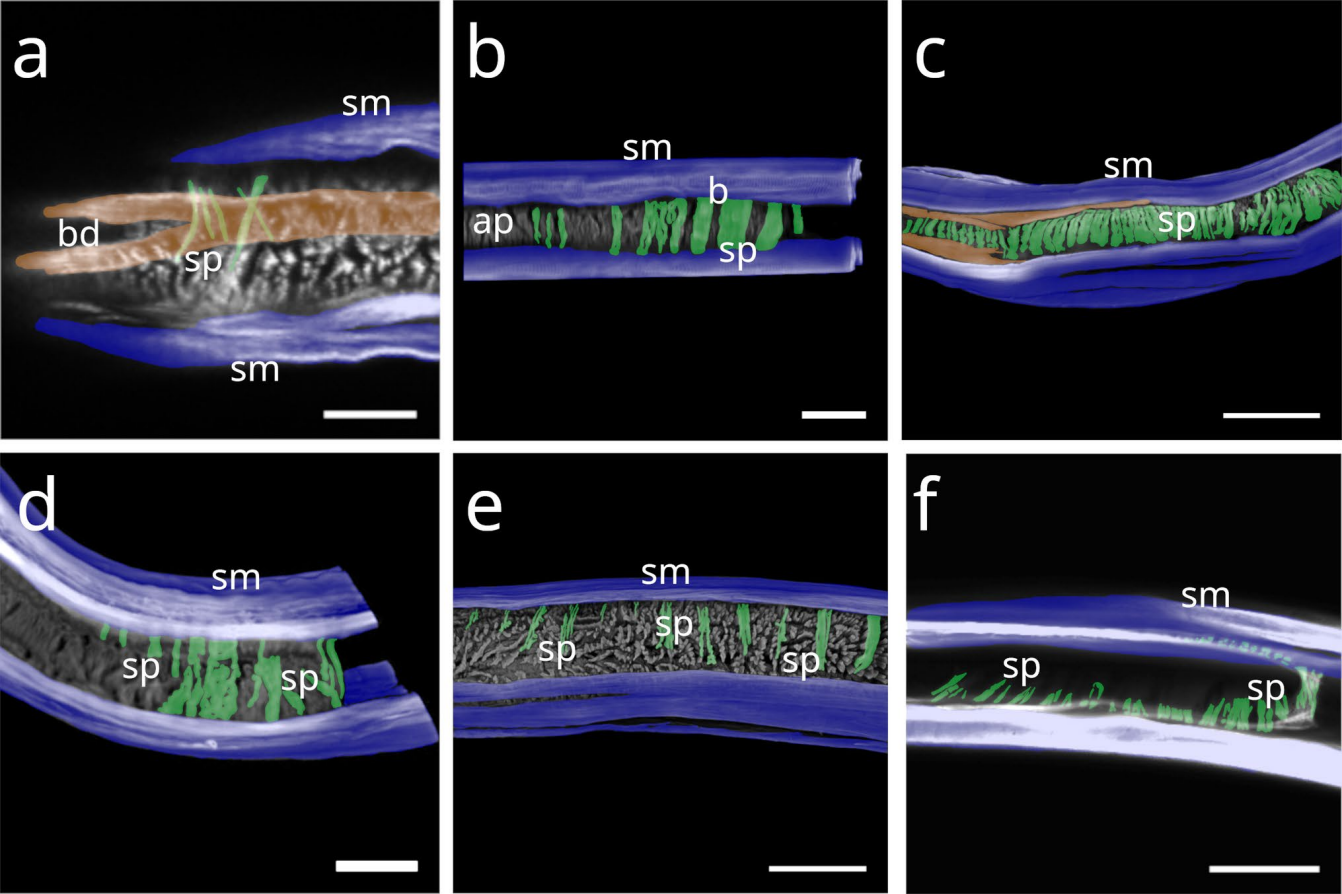
Spiral musculature of Chromadorida. CLSM micrographs. Buccal dilators in brown, somatic muscles in blue, spiral musculature in green. Anterior to the left (**a**) *Chromadorina* sp. Anterior pharynx. (**b**) *Chromadorina* sp. Posterior pharynx. (**c**) *Paracyatholaimus* sp. Pharynx area (**d**) *Pomponema* sp. Posterior pharynx. (**e**) *Synonchiella* sp. Posterior pharynx. (**f**) *Marylynnia* sp. Posterior pharynx area. Pharynx was removed using mechanical pressure. The musculature surrounding the pharynx still present. Abbreviations ap anterior pharynx, b base of the pharynx, bd buccal dilators, sm somatic musculature, sp spiral muscle. Scalebars (a) 10 µm (b) 20 µm (c) 40 µm (d) 50 µm (e) 40 µm, (f) 40 µm

In *Chromadorina* sp., the spiral muscle is present at both the anterior (Fig. 18a) and posterior ends of the pharynx (Fig. 18 b), consisting of few apparently continuous coils.

In *Paracyatholaimus* sp., dense spiral musculature surrounds the pharynx (Fig. 18 c).

In *Pomponema* sp., the posterior pharynx is encircled by few apparently continuous coils (Fig. 18 d).

In *Synonchiella* sp., spiral musculature is limited to the posterior pharynx, where few brace-like muscles were observed (Fig. 18 e).

In *Marylynnia* sp., the pharynx was removed during preparation. The remaining muscle sheath, which surrounds the posterior pharynx in vivo, bilaterally, is still visible and in direct contact with the somatic musculature (Fig. 18 f).

#### Enoplida

No spiral musculature was detected in *Thalassironus* sp., *Eurystomina* sp., *Enoploides* sp., or *Mesacanthion* sp.

### Statistical analysis of the Stilbonematinae spiral muscle

**Fig. 19:**
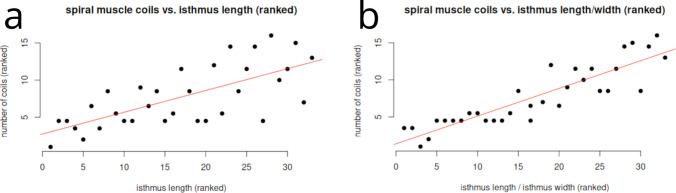
Plot of the Stilbonematinae spiral muscle. The number of coils around the anterior pharynx (in case of a two-part pharynx) or isthmus (in case of a three-part pharynx) vs. (**a**) length of the anterior pharynx/isthmus (**b**) slenderness (length divided by width) of the anterior pharynx/isthmus. Data points shown were transformed into ranks for Spearman correlation coefficient

Statistical analysis showed that the number of spiral muscle coils in Stilbonematinae is more strongly associated with pharyngeal slenderness than with pharyngeal length. Specifically, the number of coils around the isthmus (in three-part pharynges) or the anterior pharynx (in two-part pharynges) exhibited a very strong positive correlation with slenderness (Spearman’s r = 0.92, p < 0.05, df = 31). In contrast, the correlation with pharyngeal length, although still significant, was weaker (Spearman’s r = 0.70, p < 0.05, df = 31). These findings indicate that the degree of spiral coiling is primarily determined by the relative slenderness of the pharynx rather than its absolute length (Table 1, Fig. 19).

## Discussion

### *Buccal dilator musculature (*Fig. 20, 23*)*

**Fig. 20:**
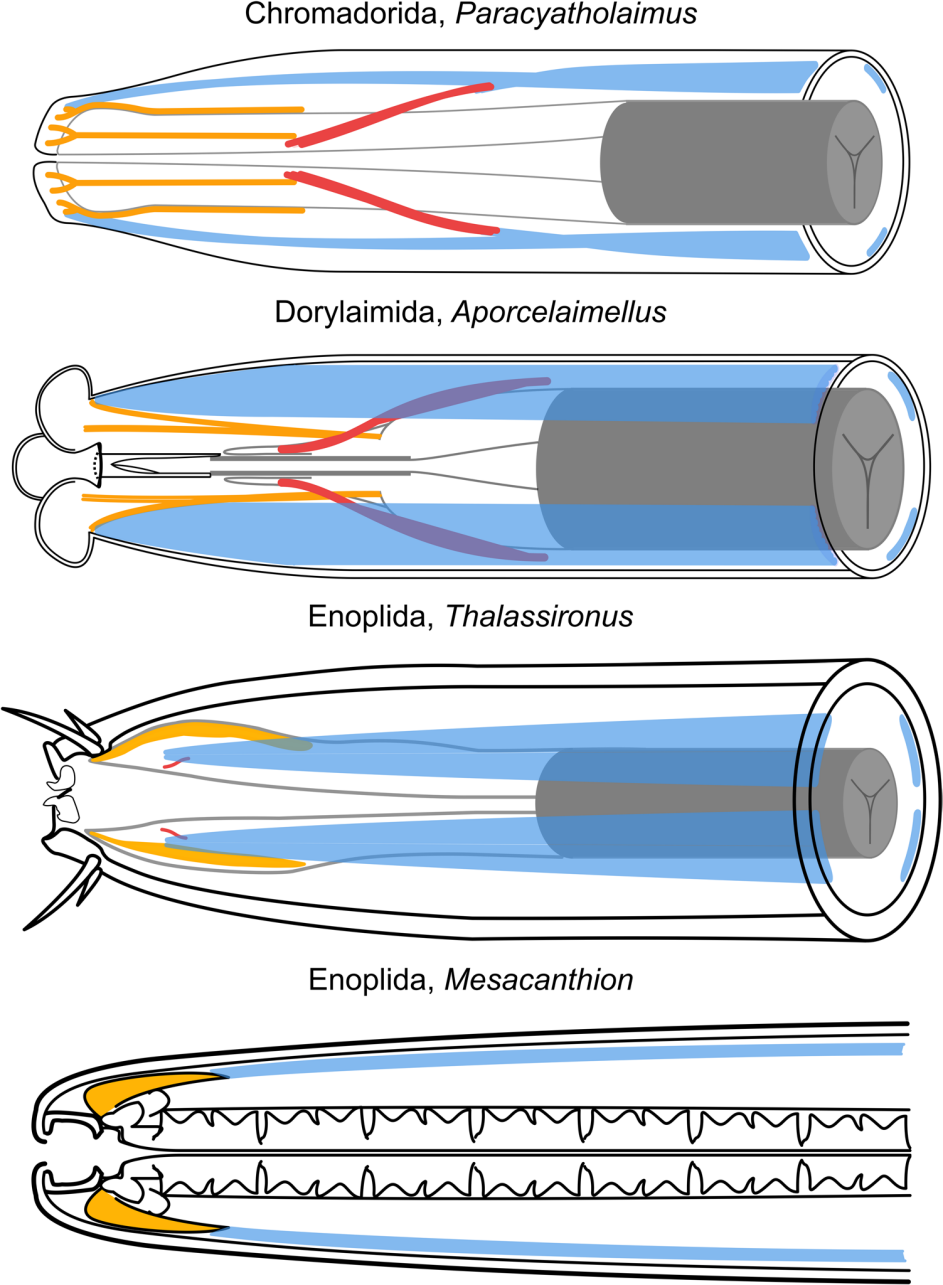
Schematic comparison of the buccal dilators, protractors of Chromadoria, Dorylaimia and Enoplia and the retractor muscles of Chromadoria and Dorylaimia. Buccal dilators (Chromadoria) and protractors (Dorylaimia), and modified pharynx muscles (Enoplia) in orange, somatic musculature in blue, somato-pharyngeal muscles in red (purple if covered by somatic musculature). Anterior to the left

The buccal dilators of Chromadorida and Desmodorida represent extensions of the somatic musculature, as indicated by their delicate continuity with the body wall muscles. In both groups, the buccal dilators extend from the pharyngeal surface in subdorsal, subventral, dorsal, and ventral positions towards the anterior extremity. We regard these muscles as homologous. Although the connection between buccal dilators and somatic musculature has been reduced in some Desmodorida, it remains present in certain species and individuals of Chromadorida and Stilbonematinae (Desmodoridae).

To further evaluate homology among Chromadoria, Dorylaimia, and Enoplia, comparisons with representatives of Enoplia and Dorylaimia are essential. In Dorylaimida, part of the stomatal musculature consists of “*dilatatores buccae*”. These differ fundamentally from the buccal dilators of Chromadoria described here, since they extend from the anterior body wall musculature to the stomatal walls and are not associated with the pharynx (Peña-Santiago, 2021). Muscles more comparable to Chromadorian buccal dilators are the *odontostyle protractors*, or “protractor muscles of the stylet.” Accordingly, our assessment of homology focuses on the relationship between odontostyle protractors of Dorylaimida and buccal dilators of Chromadoria.

Odontophore protractors consist of four muscles (each composed of two bundles, thus eight bundles in total) arranged in subdorsal and subventral positions (Peña-Santiago, 2021).

For *Aporcelaimellus obtusicaudatus* Bastian, 1865 (syn. *Dorylaimus obtusicaudatus*; Dorylaimida, Dorylaimina), eight protractor muscles extend from the anterior pharynx to the cuticle of the anterior extremity, with partial insertion into the body wall (“protractor muscles” in Lippens et al., 1974; “somato-esophageal muscles” in Chitwood & Chitwood, 1977).

In *Xiphinema* (Dorylaimida, Dorylaimina), six to eight muscles spiral around the odontophore base (Wright et al., 1983), which is derived from pharyngeal tissue (Bird & Bird, 1991; Peña-Santiago, 2021). These extend anteriorly towards the cheilostome (Munn & Munn, 2002) and connect to the anterior somatic musculature (Wright et al., 1983).

Similarly, *Longidorus elongatus* Micoletzky, 1921 (Dorylaimida, Dorylaimina) has eight odontophore protractors extending from the odontophore base to the buccal capsule, each originating from a quadrant of somatic muscles (Taylor et al., 1970).

In *Calodium hepaticum* Moravec, 1982 (Trichinellida, Trichinellina; syn. *Capillaria hepatica*), two buccal dilator muscles (termed “*posterior dilator muscles*”) extend from the anterior pharynx (in lateral position) anteriorly towards the body wall, bifurcate into two slips that connect sublaterally (Wright, 1974). Although the muscles fuse and attach to the lateral pharynx surface, we assume that they are derived from anterior subdorsal and subventral somatic muscles.

In *Nygolaimus* (Dorylaimida, Nygolaimina), protractor muscles extend from the anterior pharynx to the anterior body wall (Coomans & De Coninck, 1963; Munn & Munn, 2002).

Comparable conditions are also seen in Mononchida. In *Mononchulus nodicaudatus* Cobb, 1918 (Mononchida, Bathyodontina), six labial muscles extend from the anterior pharynx to the lip region in dorsal, ventral, subdorsal, and subventral positions; these bifurcate anteriorly and insert at the extremity (Coomans & Loof, 1970).

An identical arrangement is reported in *Mylonchulus dentatus* Jairajpuri, 1970 (Mononchida, Mononchina; Jairajpuri & Azmi, 1978).

In *Tigronchoides amiciae* Coomans & Lima, 1965 (Mononchida, Mononchina; syn. *Anatonchus amiciae*), protractors are in direct contact with the anterior end of the somatic muscles (Coomans & Lima, 1965).

Odontophore protractors and labial muscles of Dorylaimida and Mononchida exhibit striking similarities to the buccal dilators of Chromadorida and Desmodorida. Chromadoria possess subventral, subdorsal, dorsal, and ventral buccal dilators. Mononchida exhibit the same configuration, whereas Dorylaimida lack dorsal and ventral protractors. We infer that dorsal and ventral buccal dilators were lost in Dorylaimida but retained in Mononchida.

In all cases, the muscles extend from the anterior pharynx to the anterior extremity. In Dorylaimida, the odontophore (posterior odontostyle) to which these muscles attach is itself derived from pharyngeal tissue. Furthermore, their fine structure resembles that of somatic muscles (Peña-Santiago, 2021). Taken together, these observations strongly support homology between Chromadorian buccal dilators and the protractor/labial muscles of Dorylaimida and Mononchida, respectively, all derived from the somatic musculature.

The situation in Enoplia is more complex. Patterns of perioral muscle attachment can be grouped into three types:

- **Direct pharynx–cephalic framework connection.** Found in *Mesacanthion*, *Enoploides* (Enoplida, Enoplina), and *Thalassironus* (Enoplida, Ironina), as well as *Rhabdodemania* (Triplonchida, Tobrilina; Hope, 1988) and several Trichodoridae (Triplonchida, Diphtherophorina; Karanastasi et al., 2004). Van der Heiden (1974) described additional *sectorial longitudinal protractors* and *tooth muscles* in Ironidae, extending from the distal anterior pharynx to the extremity. Similar muscles were reported for *Dolicholaimus* (Ironidae, Thalassironinae).
- **Specialized somatic–pharynx connection.** In *Eurystomina* (Enoplida, Oncholaimina) the somatic muscles give rise to seven branched buccal dilators, with an anterior and posterior part: the anterior branch extends along the anterior pharynx surface to the cephalic framework, whereas the posterior branch progresses along the pharynx surface posteriorly.
- **Tendon-based system.** In *Deontostoma* (Ironina, Leptosomatidae), the anterior pharynx contains a tendon anchoring multiple labial dilators along its course (Hope, 1982). The tendon traverses the pharynx and inserts at the anterior cuticle, such that contraction of associated muscles is assumed to expand the buccal cavity.

Maximum parsimony indicates that the ancestral Enoplian plan was of the first type, retained in both Enoplida (except Oncholaimina) and Triplonchida. The second type in Oncholaimina represents a secondary modification of parts of the somatic musculature into protractors. Notably, Oncholaimina differ from Chromadoria and Dorylaimia in possessing a pair of ventral buccal dilators (each originating from a subventral somatic muscle), whereas Chromadoria and Dorylaimia have a single ventral dilator formed from both subventral somatic muscles. This distinct difference suggests that *Eurystomina* buccal dilators evolved independently and are not homologous to those of Chromadoria/Dorylaimia.

The identical positions of (i) protractor/labial muscles in Dorylaimia, which extend from the basal ECM of the anterior pharynx to perioral sites, and (ii) buccal dilators in basally branching Chromadoria (Chromadorida and Desmodorida), strongly suggest a synapomorphy between Dorylaimia and Chromadoria.

### *Somato-pharyngeal musculature (*Fig. 20, 23*)*

Our morphological data demonstrate an identical pattern of somato-pharyngeal musculature in Chromadorida and Desmodorida. These muscles extend from the somatic musculature anteriorly and insert either (i) at the anterior pharynx (anterior somato-pharyngeal muscles) or (ii) at the posterior pharynx (posterior somato-pharyngeal muscles).

In other Chromadoria, such anterior muscles have so far been reported only for *Siphonolaimus* (Monhysterida, Linhomoeina), in which fibres extend from the body wall musculature to the anterior pharynx and further to the anterior extremity (Chitwood & Chitwood, 1977). The authors did not discriminate between “somato-pharyngeal” and “buccal dilator” muscles as we do in this paper.

Within Dorylaimina, somato-pharyngeal muscles have been described for *Aporcelaimellus obtusicaudatus* (Dorylaimida, Dorylaimina; Chitwood & Chitwood, 1977, as *Dorylaimus obtusicaudatus*). These “posterior somato-esophageal” muscles consist of four bands corresponding to the anterior somato-pharyngeal muscles described in this study. They extend from the body wall anteriorly towards the pharynx, close to the stylet protractors.

In Enoplia, posterior somato-pharyngeal muscles have been documented in several taxa. In *Enoplus communis* Bastian, 1865 (Enoplida, Enoplina), eight such muscles extend from the body wall posteriorly towards the pharynx–intestine junction (Chitwood & Chitwood, 1977; De Man, 1886). Comparable conditions are present in *Oncholaimus* (Enoplida, Oncholaimina; De Man, 1886), *Metoncholaimus* (Enoplida, Oncholaimina; Chitwood & Chitwood, 1977), *Eurystomina* (Enoplida, Oncholaimina), and *Thalassironus* (Enoplida, Ironina). In *Tuerkiana strasseni* Platonova, 1970 (Enoplida, Ironina; syn. *Thoracostoma strasseni* Türk, 1903), four muscles extend posteriorly from the somatic musculature and attach to the pharynx anterior to the peripharyngeal nerve ring (Chitwood & Chitwood, 1977).

In Stilbonematinae, somato-pharyngeal muscles also originate from the somatic musculature. Their anterior somato-pharyngeal muscles (this work) resemble the posterior portion of the “somato-esophageal muscles” described by Chitwood and Chitwood (1977) in *Siphonolaimus* and *Aporcelaimellus obtusicaudatus* (see above), as well as the odontostyle retractors of Dorylaimida. The anterior portion of those “somato-esophageal” muscles, however, resembles the buccal dilators of Stilbonematinae (this work) and the protractor/labial muscles of Dorylaimia.

The anterior somato-pharyngeal muscles of Stilbonematinae also resemble those reported for *Tuerkiana strasseni*. In both cases, they arise from the body wall musculature and insert into the pharynx anterior to the peripharyngeal nerve ring. Although in Stilbonematinae the buccal dilators and somato-pharyngeal muscles appear continuous at the first glance, they are clearly distinct. This raises the possibility that the seemingly continuous “somato-esophageal” muscles of *Siphonolaimus* and *Aporcelaimellus* (see above) should be re-examined to determine whether they too are composed of two distinct muscle groups. We strongly assume that this is the case.

In summary, somato-pharyngeal muscles of basally branching Chromadorida and Desmodorida, the odontophore retractors of Dorylaimia, and the retractor muscles of Enoplia share an identical origin: they arise from the somatic musculature and attach to the pharynx. We therefore conclude that these muscles are homologous across the three subclasses.

### *The pharynx spiral musculature of Nematoda *(Fig. 21, 23)

**Fig. 21:**
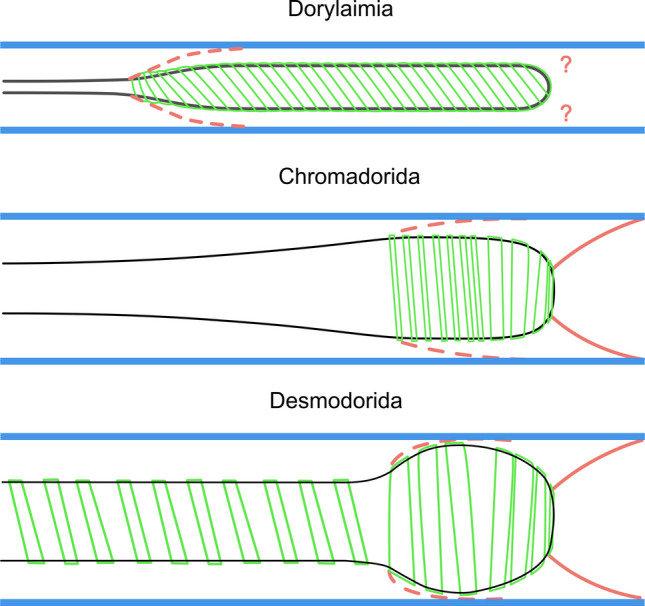
Schematic drawings of the spiral musculature of Dorylaimia and Chromadoria. Somatic musculature in blue, somato-pharyngeal muscles in red, spiral musculature in green

To our knowledge, a spiral musculature around the pharynx has not previously been reported in Chromadoria. Its obscurity when using standard light microscopy for taxonomic work likely explains why it was overlooked. Chitwood and Chitwood (1977) mentioned only a semicuticular membrane as a frequent external covering of the nematode pharynx, without further detail. When spiral musculature was occasionally observed in Dorylaimia, it was sometimes dismissed as a fixation artefact; only later was the muscular nature recognized (Siddiqi, 1968).

To this day, reports of spiral muscles surrounding parts of the pharynx are restricted to the subclass Dorylaimia. The most prominent example is the muscular sheath around the posterior pharynx in Belondiridae (Dorylaimida, Belondiroidea), which is robust and easily observed (Chitwood & Chitwood, 1977; Hechler, 1969; Peña-Santiago, 2014). In this group, the spiral muscles encircle the posterior pharynx in dextral, ventral, or straight orientations.

Other examples occur in *Calodium hepaticum* (Trichinellida, Capillariidae) and *Trichinella spiralis* (Trichinellida, Trichinellidae), both of which possess muscular sheaths around the posterior pharynx (Beckett & Boothroyd, 1961; Wright, 1974).

Several families within Nygolaimina (e.g. Aetholaimidae, Nygellidae, Nygolaimellidae, Nygolaimidae) also show delicate sheaths around the posterior pharynx, sometimes conspicuously spiral (Peña-Santiago, 2014).

In *Xiphinema* (Dorylaimina, Longidoridae), cellular membranes around the anterior pharynx develop into distinct longitudinal muscles along the posterior pharynx (Roggen, 1979; Wright, 1965).

In *Longidorus elongatus* Micoletzky, 1921 (Dorylaimina, Longidoridae), a longitudinal muscle (U-shaped in cross-section) encircles the pharynx at mid-length (Taylor et al., 1970). At the level of the posterior bulb, this muscle gives rise to four connections with the somatic musculature, a sphincter, and three sets of equidistant longitudinal muscles around the bulb — all derived from somatic musculature.

Our study documents several instances of peri-pharyngeal spiral muscles in Chromadorida, encircling the posterior bulb, as well as in Desmodorida and *Paracyatholaimus* (Chromadorida), where they also surround the anterior pharynx. In both Chromadoria and Dorylaimia, these spiral muscles are derivatives of the somatic musculature.

Taken together, the presence of posterior pharyngeal spiral musculature in Chromadorida, Dorylaimida, and Trichinellida suggests homology. This represents a potential synapomorphy linking Chromadoria and Dorylaimia.

### *Functional assessment of the buccal dilators and somato-pharyngeal muscles in Stilbonematinae (*Fig. 22*)*

**Fig. 22:**
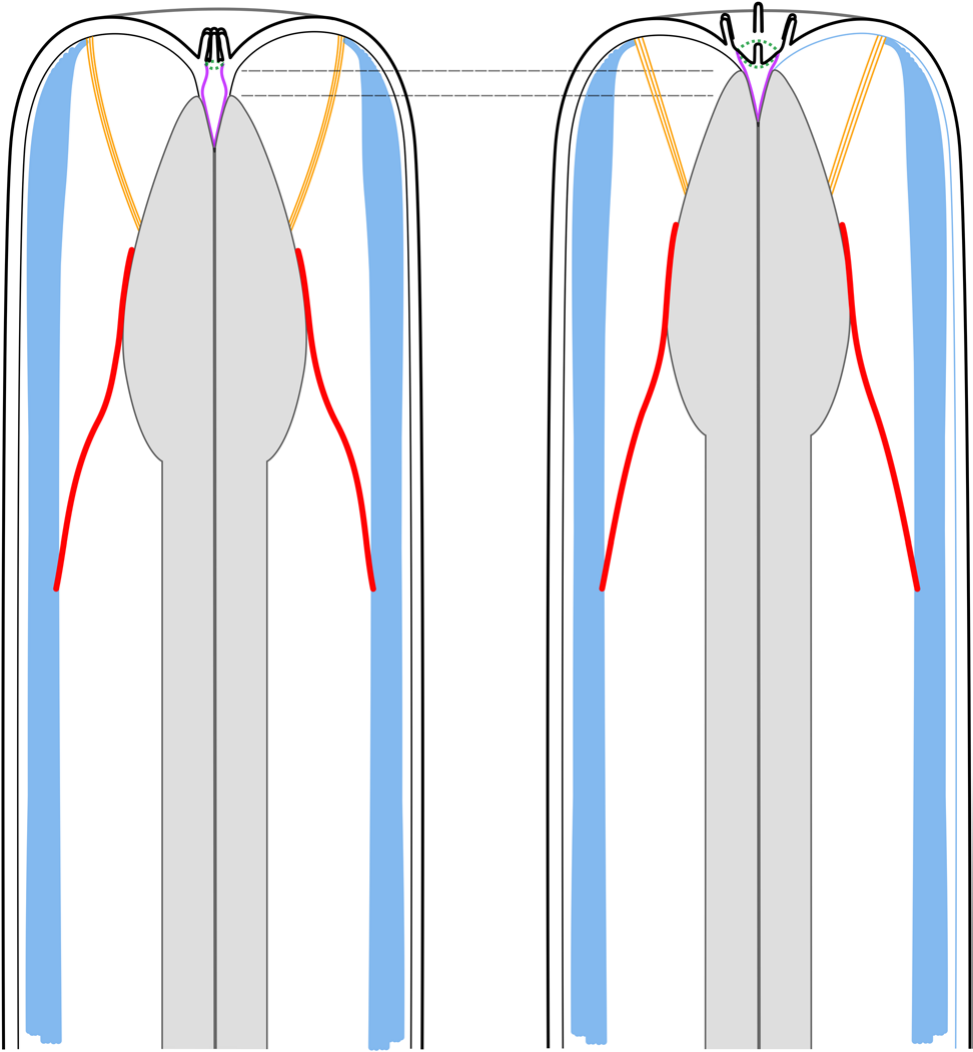
Hypothetical scenario of the usage of buccal dilators and somato-pharyngeal muscles using *Catanema* (Stilbonematinae) as an example. Schematic drawing. Somatic musculature in blue, somato-pharyngeal muscles in red, buccal dilators in orange, buccal cavity in magenta, mouth opening as a dashed green line, pharynx in light grey. Anterior to the top. (left) Mouth closed and pharynx retracted. (right) Mouth opened and pharynx protracted

Neither buccal dilators nor somato-pharyngeal muscles have previously been described in Stilbonematinae, and direct feeding observations are lacking. Consequently, their functional role must be inferred solely from morphology, under the assumption that these muscles are active.

Since Stilbonematinae lack buccal armature (no teeth or spear), the buccal dilator muscles cannot be involved in armature movement. Instead, they extend from the anterior pharynx to perioral sites along the subdorsal, subventral, dorsal, and ventral axes. Their insertions do not correspond to the positions of inner or outer labial papillae. Notably, nematodes lack dorsal and ventral papillae altogether, and the insertions lie more distally at the terminal extremity than the inner labial papillae, which retract into the mouth opening to form a cone-like covering in preserved specimens. An association with only the subdorsal and subventral outer labial papillae also seems unlikely, as there are no reports of active outer labial papilla movement in nematodes.

We therefore propose that contraction of the buccal dilators pulls the pharynx anteriorly towards the mouth opening. This action likely shifts the buccal cavity forward, partly everting it. The mouth opening would widen, and the inner labial papillae would be displaced passively, spreading outward in a circular arrangement. Such a sequence of positional changes is assumed to occur during feeding.

The somato-pharyngeal muscles, in turn, are likely antagonistic to the dilators. By contracting, they would pull the pharynx posteriorly back to its resting position, restoring the cone-like arrangement of the inner labial papillae and facilitating passive closure of the mouth opening (Fig. 22).

### *Functional assessment of the spiral musculature in Stilbonematinae and statistical support (*Fig. 19*)*

In Stilbonematinae, the number of muscular coils around the pharynx shows a clear statistical correlation with the length of the cylindrical anterior pharynx or, in three-part pharynges, with the isthmus. There is, however, a higher positive correlation with pharyngeal slenderness: the more slender the pharynx or isthmus, the greater the number of muscular coils, and vice versa. This relationship supports Roggen’s (1979) pharynx model.

According to Roggen, when the lumen of a cylindrical pharyngeal region is very narrow, hydraulic resistance increases to the point that the cylindrical portion alone cannot generate sufficient injection pressure to move food into the gut. Within Stilbonematinae, the genera *Catanema* and *Robbea* possess the most slender isthmus, and correspondingly the highest number of muscular coils around it. These genera also exhibit a distinct set-off corpus (Pröts et al., 2024). Roggen’s model predicts that a spherical bulb can generate twice the injection pressure of a cylindrical pharyngeal region of equivalent volume and lumen diameter. Thus, a swollen corpus should augment injection pressure, facilitating food transport into the intestine.

We therefore infer that in *Catanema* and *Robbea*, the combination of numerous muscular coils around the slender isthmus and the presence of a swollen anterior corpus generates the necessary pressure to transport food efficiently through the pharynx. In addition, because the posterior bulb is also encircled by muscle, this likely both drives forward movement of gland secretions and provides additional pumping pressure, thereby ensuring efficient passage of food into the intestine.

Roggen’s model also explains the absence of spiral musculature in infraorders such as Tylenchomorpha. These nematodes possess a three-part pharynx with a slender procorpus and isthmus, neither of which bears spiral muscles. Instead, they possess a prominent metacorpus at mid-pharynx. As (semi-)passive feeders, these animals exploit the turgor pressure of host cells (pierced by the stylet) as a source of injection pressure for the anterior pharynx (Doncaster, 1966). In this context, the metacorpus likely provides relatively less suction but greater injection pressure, compensating for the resistance of the slender procorpus and isthmus.

### *Conclusion: phylogenetic relationships between Chromadoria and Dorylaimia, and outlook *(Fig. 23)

The morphological data presented here are highly relevant for resolving the relationships among Chromadoria, Dorylaimia, and Enoplia. While recent molecular analyses have already suggested a closer affinity between Chromadoria and Dorylaimia (Ahmed & Holovachov, 2021; Ahmed et al., 2022; Smol et al., 2020), our results provide strong morphological evidence that supports these hypotheses.

**Fig. 23:**
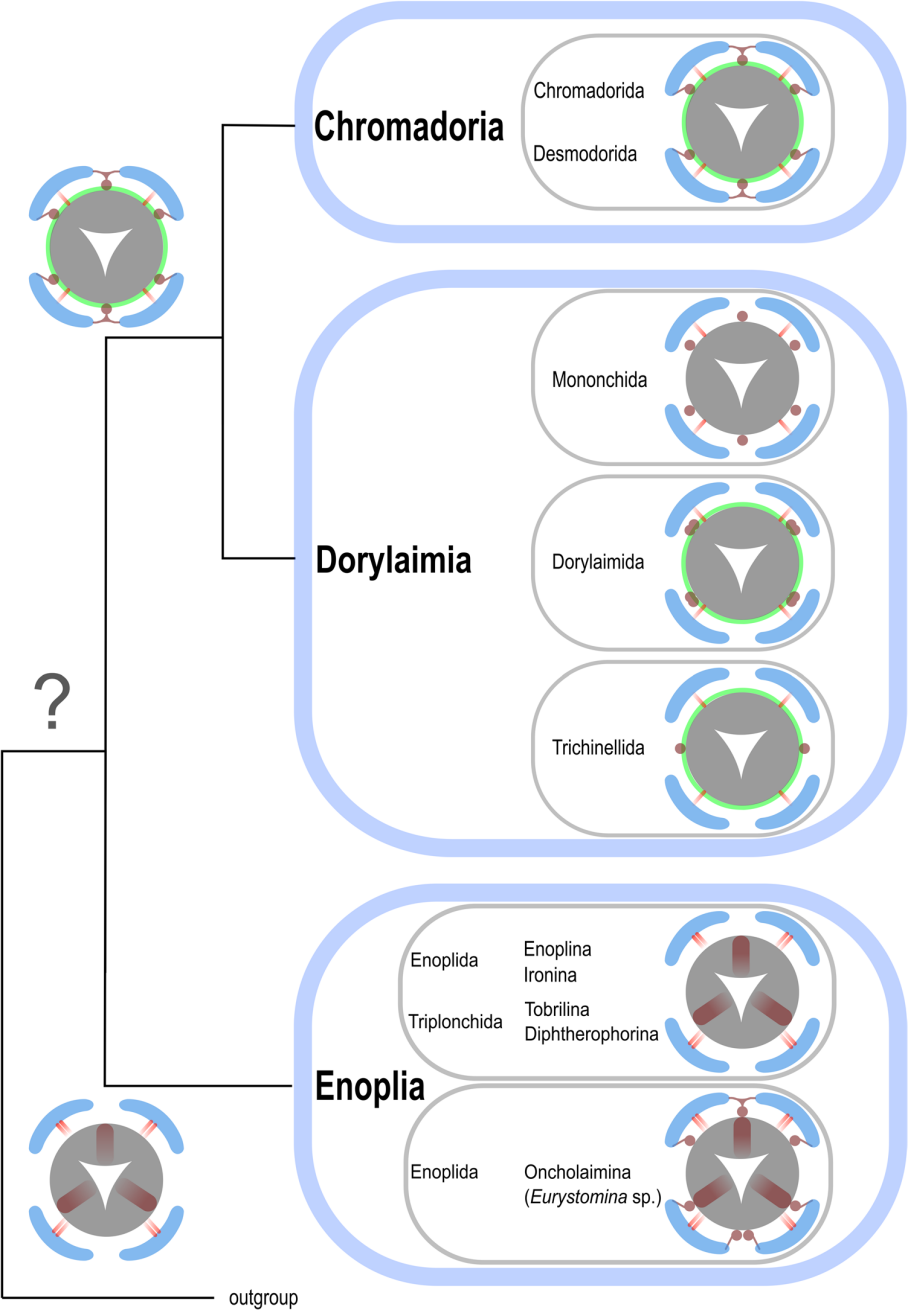
Distribution of pharynx associated musculature of Chromadoria, Dorylaimia and Enoplia. Buccal dilators/protractors in brown, somatic musculature in blue, somato-pharyngeal muscles in red, spiral musculature in green. Opacity gradient of a muscle indicates the relative position from anterior (full opacity) to posterior (low opacity). The depicted protractors of Enoplia indicate the fusion of the anterior pharynx with the cephalic framework

Pharynx-associated musculature is functionally critical for nematode feeding and therefore unlikely to undergo rapid and random evolutionary change. As such, these features represent robust characters for phylogenetic inference. Three independent lines of evidence in sum suggest a sister-group relationship between Chromadoria and Dorylaimia:

- **1. Buccal dilators.** Both subclasses share the same attachment pattern, with muscles originating from somatic musculature, extending from the anterior pharynx, and inserting at the anterior extremity. As already mentioned above, the similar situation found in *Eurystomina* sp. (Enoplida, Oncholaimina) is homoplastic.
- **2. Somato-pharyngeal (or retractor) muscles.** In both Chromadoria and Dorylaimia, somatic musculature gives rise to muscles that extend anteriorly and attach to the anterior pharynx or odontophore. Although comparable muscles in Enoplia are oriented posteriorly and insert at the pharynx base (here they functionally resemble protractors), they too originate from the somatic musculature. Thus, they are homologous across all three subclasses. However, because they are present in all three groups, somato-pharyngeal muscles are not useful for resolving relationships among them.
- **3. Spiral musculature.** In Chromadorida (the most basally branching group of Chromadoria), spiral muscles surround the posterior bulb in the same position as spiral muscles described for several orders and superfamilies of Dorylaimia. The same pattern of these modified somatic muscles strongly suggests homology and represents an additional synapomorphy linking the two subclasses.

Taken together, these three muscle systems demonstrate consistent homology between Chromadoria and Dorylaimia, supporting the hypothesis that they are sister groups.

Finally, just as our study uncovered previously overlooked spiral musculature in Chromadorida and Desmodorida, we expect that spiral muscles are also more widespread in Dorylaimia than currently reported. Future ultrastructural and/or immunohistochemical investigations are therefore likely to reveal additional cases of spiral musculature, further strengthening the morphological evidence for this relationship.

## Data Availability

Data is available from the corresponding author upon reasonable request.
